# The upper respiratory tract microbiota of healthy adults is affected by *Streptococcus pneumoniae* carriage, smoking habits, and contact with children

**DOI:** 10.1186/s40168-023-01640-9

**Published:** 2023-09-02

**Authors:** A. Cristina Paulo, João Lança, Sónia T. Almeida, Markus Hilty, Raquel Sá-Leão

**Affiliations:** 1 Instituto de Tecnologia Química E Biológica António Xavier, Universidade Nova de Lisboa, Oeiras, Portugal; 2 Faculty of Medicine, Institute for Infectious Diseases, University of Bern, Bern, Switzerland

**Keywords:** Microbiota, Nasopharynx, Oropharynx, *Streptococcus pneumoniae*, Healthy adults

## Abstract

**Background:**

The microbiota of the upper respiratory tract is increasingly recognized as a gatekeeper of respiratory health. Despite this, the microbiota of healthy adults remains understudied. To address this gap, we investigated the composition of the nasopharyngeal and oropharyngeal microbiota of healthy adults, focusing on the effect of *Streptococcus pneumoniae* carriage, smoking habits, and contact with children.

**Results:**

Differential abundance analysis indicated that the microbiota of the oropharynx was significantly different from that of the nasopharynx (*P* < 0.001) and highly discriminated by a balance between the classes Negativicutes and Bacilli (AUC of 0.979). Moreover, the oropharynx was associated with a more homogeneous microbiota across individuals, with just two vs*.* five clusters identified in the nasopharynx. We observed a shift in the nasopharyngeal microbiota of carriers vs*.* noncarriers with an increased relative abundance of *Streptococcus*, which summed up to 30% vs*.* 10% in noncarriers and was not mirrored in the oropharynx. The oropharyngeal microbiota of smokers had a lower diversity than the microbiota of nonsmokers, while no differences were observed in the nasopharyngeal microbiota. In particular, the microbiota of smokers, compared with nonsmokers, was enriched (on average 16-fold) in potential pathogenic taxa involved in periodontal diseases of the genera *Bacillus* and *Burkholderia* previously identified in metagenomic studies of cigarettes. The microbiota of adults with contact with children resembled the microbiota of children. Specifically, the nasopharyngeal microbiota of these adults had, on average, an eightfold increase in relative abundance in *Streptococcus* sp., *Moraxella catarrhalis*, and *Haemophilus influenzae,* pathobionts known to colonize the children’s upper respiratory tract, and a fourfold decrease in *Staphylococcus aureus* and *Staphylococcus lugdunensis*.

**Conclusions:**

Our study showed that, in adults, the presence of *S. pneumoniae* in the nasopharynx is associated with a shift in the microbiota and dominance of the *Streptococcus* genus. Furthermore, we observed that smoking habits are associated with an increase in bacterial genera commonly linked to periodontal diseases. Interestingly, our research also revealed that adults who have regular contact with children have a microbiota enriched in pathobionts frequently carried by children. These findings collectively contribute to a deeper understanding of how various factors influence the upper respiratory tract microbiota in adults.

Video Abstract

**Supplementary Information:**

The online version contains supplementary material available at 10.1186/s40168-023-01640-9.

## Background

The microbiota of the human upper respiratory tract (URT) has an important role in human health since it modulates the colonization of commensal bacteria and provides colonization resistance against pathogens [1]. The URT comprises several structures, among which the nasopharynx and the oropharynx are distinctive, as they are the preferential niches of important human pathobionts, including *Streptococcus pneumoniae* (or pneumococcus). Pneumococcus is a gram-positive facultative anaerobe that is known to be the main cause of bacterial respiratory infections worldwide [2]. Risk groups for pneumococcal disease include young children, the elderly, and immunocompromised individuals of all ages [3]. Colonization is mostly asymptomatic and is very frequent in children under 5 years of age, in which it is often higher than 50% [4–6]. In contrast, in adults, it has been reported as being between 20 and 40% [7–9]. Two important factors that contribute to increased pneumococcal colonization and persistence in adults include contact with children and smoking [9–12]. To what extent the microbiota composition is a risk factor for pneumococcal colonization has not been explored. Nonetheless, to the best of our knowledge, while there are studies in children, the microbiota of the nasopharynx and oropharynx remains poorly characterized since the few microbiota studies of the upper respiratory tract in adults have been associated with disease status [13–16].

Understanding the factors that shape and characterize a healthy microbiota is a fundamental step in strategies aimed at promoting a healthy state, for example, through the use of live biotherapeuticals [17].

Here, we comprehensively analyzed the composition of the nasopharyngeal and oropharyngeal microbiota of immunocompetent healthy adults aged between 25 and 50 years old. The specific aims of our study were (i) to compare the microbiota of pneumococcal carriers vs. noncarriers in the nasopharynx and oropharynx and (ii) to understand how individual characteristics such as age, sex, contact with children, and smoking habits shape the microbiota of the nasopharynx and oropharynx.

## Methods

### Study population and study design

A case–control study was designed with the aim of evaluating potential differences between the nasopharyngeal and oropharyngeal bacterial microbiota of immunocompetent healthy adults colonized with *S. pneumoniae* (cases) and noncolonized individuals (controls).

The study was nested in a prospective 6-month longitudinal study that aimed to characterize the dynamics of *S. pneumoniae* colonization in healthy adults [9]. The original study was conducted between February 2015 and December 2016 and enrolled 87 immunocompetent adults aged between 25 and 50 years old living in the Lisbon metropolitan area, Portugal. Detailed information about sample collection and study design is described in the supplementary information (“Sample collection”) and in Almeida et al. [9]. Briefly, nasopharyngeal and oropharyngeal samples were collected using appropriate swabs and were immediately stored in STGG medium. All samples were kept at − 80 °C. The presence of pneumococci was screened by classical culture based methods and real-time PCR targeting the genes *lytA* and *piaB* [9].

The study was approved by the ethical committee of Instituto de Higiene e Medicina Tropical, Universidade Nova de Lisboa, and was registered at the National Commission of Data Protection (ref. 3803/2014). Signed informed consent was obtained from all participants; samples and questionnaires were processed anonymously.

In the current study, cases were defined as individuals who were found to carry pneumococci in the nasopharynx and/or oropharynx at a minimum of three time points at least 1 month apart from each other. Controls were defined as individuals who were sampled at least five times (1 month apart from each other) during the 6-month period and were never colonized with pneumococci. For both cases and controls, samples collected within 1 month of antibiotic use were excluded. For both cases and controls, three samples *per* individual were selected for analyses. The three samples were, as much as possible, distant in time and covered different seasons.

### DNA extraction

Nasopharyngeal and oropharyngeal samples were maintained at − 80 °C in STGG. Samples were thawed on ice, and for each sample, 200 μL was pipetted and added to 200 μL of lysis buffer (MagNA Pure Compact Nucleic Acid Isolation Kit, Roche Diagnostics, GmbH). Samples were incubated at 37 °C for 20 min. DNA extraction was performed with the MagNA Pure Compact System (Roche) according to the manufacturer’s instructions. For every run, water samples (used as negative controls) were extracted in parallel. DNA was stored at − 20 °C. Water samples were used as technical negative controls and were processed in parallel with biological samples to control for potential contaminations arising during manipulation and testing.

### Total bacterial load quantification

To prepare a standard curve for total bacterial load quantification, the method described by Bogaert et al*.* [18] was followed with some modifications. Representative strains of eight bacterial species that commonly colonize the upper respiratory tract were used: *Corynebacterium accolens*, *Dolosigranulum pigrum*, *Haemophilus influenzae*, *Moraxella catarrhalis*, *Streptococcus mitis*, *Streptococcus oralis*, *S. pneumoniae*, and *Streptococcus pseudopneumoniae*. First, serial dilutions of the frozen stocks of each strain were performed to quantify the bacterial load. Except for *H. influenzae*, which was cultured on chocolate agar, all other species were cultured on blood agar plates. *Corynebacterium accolens* cultures were incubated overnight at 37 °C in anaerobic conditions; *Dolosigranulum pigrum* cultures were incubated overnight at 37 °C in aerobic conditions; and the remaining bacterial species were incubated overnight at 37 °C in a 5% CO_2_ atmosphere. On the following day, CFU/mL was estimated for each frozen stock. Afterwards, a mixture containing 10^4^ CFU/mL of each species was prepared. DNA extraction of the mixture was carried out as described above. DNA was quantified on a NanoDrop and used as a reference for total bacterial load quantification.

To evaluate the quality of the nasopharyngeal and oropharyngeal samples, the total bacterial load of each sample was quantified by qPCR using universal primers and probes that target the 16S rDNA gene [18]: 16Sfw-5′-CGA AAG CGT GGG GAG CAA A-3′, 16Srev-5′-GTT CGT ACT CCC CAG GCG G-3′, and FAM-ATTAGATACCCTGGTAGTCCA-MGB. qPCRs were performed in a final volume of 25 μL containing 12.5 μl of 1 × master mix (FastStart TaqMan® Probe Master, Roche), 1 μL of each primer (0.4 μM), 1 μL of probe (0.2 μM), 7 μL of H_2_O, and 2.5 μL of DNA. DNA amplification was performed in CFX96™ Real-Time System Amplification (Bio-Rad). The thermocycling conditions were 50 °C for 2 min and 95 °C for 10 min followed by 45 amplification cycles of 95 °C for 15 s and 60 °C for 1 min. In each 16S qPCR run, multiple negative controls (one per every 16 reactions) and serial dilutions (in duplicate) of the DNA extracted from the species mixture (10^0^ to 10^−5^ ng/μL) were included. The latter was included to obtain a standard curve for each qPCR.

A standard curve was considered valid when the difference between Ct values of consecutive dilutions did not exceed three Ct values and the paired results obtained for a given dilution did not exceed 0.5 Ct. Samples with a bacterial load lower than the DNA extraction negative control were considered of low quality and, whenever possible, were replaced by other samples from the same individual following the inclusion and exclusion criteria described above.

### 16S rRNA gene amplicon sequencing

For all samples, the V4 region of 16S rRNA was amplified using forward (5′-GTGCCAGCMGCCGCGGTAA-3′) and reverse (5′-GGACTACHVGGGTWTCTAAT-3′) primers previously described [19]. PCR was conducted in a final volume of 25 μL containing 10 μL of 2 × master mix, 2.5 μL of primer barcode (2 μM), 2.5 μL of universal primer (2 μM), and 10 μL of DNA. The thermocycling conditions were 94 °C for 3 min, 35 amplification cycles of 94 °C for 1 min, 50 °C for 1 min, 72 °C for 105 s, and a final extension of 72 °C for 10 min. Each sample was run in triplicate. After that, triplicates were pooled and submitted to a next-generation sequencing platform for indexing and pair-end sequencing (2 × 250 bp) on a MiSeq platform. Amplification and sequencing were performed at the Genomics Unit of Instituto Gulbenkian da Ciência.

### Bioinformatic processing

Divisive Amplicon Denoising Algorithm 2 (DADA2) [20] was used to denoise and taxonomically assign the 16S rRNA sequences following the authors’ online pipeline tutorial 1.16 (https://benjjneb.github.io/dada2/tutorial.html). DADA2 was run on R version 3.6.2 [21]. The parameters used in each step of the DADA2 workflow were those predefined and recommended in the pipeline except for the parameters that are data driven, specifically trimming and inference of error rates. Sequences were trimmed in the position in which the 25th percentile of the quality score was above 30 (see supplementary information and Fig. S1); error inference rates were calculated using the entire dataset and pooled sequences. In brief, reads were filtered and trimmed to remove sequencing errors, based on their quality scores (Phred scores) and on the identification of ambiguous bases in both forward and reverse reads. Subsequently, an estimation of the error rates (i.e., possible transitions or transversion point mutations), made by MiSeq platform, was performed. This step aimed to achieve the following: (i) infer amplicon sequence variants (ASVs) based on the estimated error rates mentioned earlier, (ii) dereplicate reads to obtain unique sequences, and (iii) remove singletons. Afterward, the forward and reverse reads were ready for merging. The final step involved identifying and removing ASVs that could potential be chimeric sequences originating from defective PCR amplification (for example, resulting from pairing of incomplete parental sequences). Taxonomy was assigned using the Silva v132 database as a reference [22].

Several additional approaches were used to remove potential contaminants by filtering ASVs and samples. First, ASVs attributed to Eukaryota and Archaea were excluded. Second, ASVs were filtered according to a frequency-based approach described by Davis et al. [23]. This approach is based on the observation that the probability of having contaminants is higher when the DNA concentration is lower. Briefly, for each ASV, a regression line was fitted to the number of reads as a function of DNA concentrations measured by 16S qPCR in each sample. If the number of reads of an ASV was observed to decrease linearly with increased DNA concentration, it was considered a contaminant and was excluded. Otherwise, it was kept in the analysis. ASVs were filtered according to their relative abundance and were kept if they were present in at least two samples, with a relative abundance within each sample higher than 0.1% [24]. Finally, samples that had fewer than 1000 reads after all ASV filters were excluded [14, 25].

For ASVs that were shown to be significantly different in the differential abundance analysis, NCBI BLAST searches were performed (using MegaBLAST) to identify the presumptive species. Species assignment was based exclusively on hits with 100%, as differences in one or more nucleotides result in assignment of different ASVs.

### Statistical analysis

All analyses described below were performed in R version 3.6.2 (Boston, MA, USA).

### Statistical analysis of the study population

The baseline characteristics of the study population and samples were stratified by the presence/absence of pneumococci. To compare characteristics between strata, the chi-square or Student’s *t*-test was used in conformity with the type of data. Bacterial DNA quantification was stratified by the presence/absence of pneumococci and by the anatomical site (oropharynx or nasopharynx). Bacterial DNA quantification, per stratum, was summarized by their geometric mean and respective standard deviation (SD). The Wilcoxon rank-sum test with continuity correction was used for multiple comparisons of groups two by two. The Benjamini–Hochberg procedure was used to control for the false discovery rate at the level of 0.05. Differences between strata were considered statistically significant if the adjusted *P-*value was < 0.05.

### Statistical analysis of nasopharyngeal and oropharyngeal microbiota profiles

The statistical analysis of the microbiota was performed using compositional data analysis methods. In brief, for each sample, count reads were normalized using the centered log_2_-ratio (CLR) transformation [26]. This transformation allows us to account for the complex compositional data structure of metagenomic studies and to reduce the likelihood of spurious correlations. The microbiome package for the CLR transformation, which replaces ASV read counts with exact zero relative abundance with a pseudocount before calculating the logarithms, was used [27].

To identify homogeneous bacterial communities in the nasopharynx and oropharynx, a hierarchical clustering approach was employed. The samples were transformed using the CLR transformation, and the Euclidean distance between samples was used for clustering. The Ward’s minimum variance method [28] was used to agglomerate samples that share similar taxonomic profiles. To determine the optimal number of clusters, the gap statistics proposed by Tibshirani et al*.* [29] were used. For cross-validation, a random forest model classifier with 500 trees was utilized. The out-of-bag error, representing the percentage of misclassified samples, was estimated, and the confusion matrix was examined to assess the degree of cluster overlap. Each cluster was characterized by the two most abundant genera it contained.

To study associations between clusters and the pneumococcal carrier state, mixed general linear models with a logit link function were used. A model was fit to each microbiota profile using the microbiota profile comprising the higher number of samples as a reference. Individuals were introduced as a random variable to account for repeated measurements. In addition, models were adjusted for sociodemographic characteristics and environmental factors (individual’s age, gender, having contact with children, smoking habits, and season in which the sample was collected). Associations between variables and the clusters were calculated using odds ratios (ORs) and corresponding confidence intervals (CIs) at 95%. A CI that did not include 1 was considered statistically significant.

### Microbiota α-diversity

The abundance-based diversity of the microbiota groups was estimated using Hill's first five numbers [30]. Hill’s numbers have a scaling parameter, known as the order of diversity (*q*), that modulates sensitivity toward more abundant or rare taxonomic units. The higher the order, the higher the importance attributed to abundant taxonomic units. The Hill numbers of orders 0, 1, and 2 are related to three popular diversity indexes known as richness, Shannon’s index, and Simpson’s index, respectively, with the advantage of having the replication principle (i.e., when doubling the number of taxonomic units in a system, the diversity is also doubled). Evenness was measured according to the steepness of the diversity profile from the Hill number of order 0 to the Hill number of order 1 (the higher the steepness, the lower the community evenness). The Hill numbers were calculated for each sample using the abundance-based estimates at the taxa level of genus and then by calculating the geometric mean for each group. Differences in Hill numbers between groups were calculated using the Mann‒Whitney test.

### Differential abundance analysis of microbiota

To evaluate if there were significant differences between the microbiota of different groups, a permutational multivariate analysis of variance (PERMANOVA) [31] implemented on the Adonis algorithm of the R Vegan package was utilized. PERMANOVA was performed on the Euclidean distance matrix with CLR-transformed read counts. To ensure the reliability of the results, the assumption of variance homogeneity was checked. If a significant effect was found, a differential abundance analysis to determine which taxa were differentially abundant between groups of samples was performed. Since the data were very sparse, we made use of a zero-inflated Gaussian mixed model (ZIGMM) [32] implemented in R with metagenomeSeq [31]. The cumulative sum scaling method was used to normalize sequence counts based on the lower-quartile abundance of features. Data were also filtered to maintain a threshold of ASVs that were present in at least 75% of the samples, a step needed to avoid unreliable fold-change estimates [32]. Only ASVs with more than 1.5 log fold-change differences and an adjusted *P-*value ≤ 0.05 were considered [31]. Volcano plots of the log_10_ of statistical significance (*P-*value) vs. log_2_ of the magnitude of change (fold-change) were used to visualize the results.

Microbial signatures, that is, groups of microbial taxa that are predictive of a phenotype of interest, were further identified using the algorithm selbal developed in R [33]. Briefly, this algorithm takes the log ratio of the geometric mean of the taxa from two groups and tests for association with the response variable by fitting a logistic model. The model that maximizes the area under the receiver operating characteristic (AUC) curve is then selected.

### Dynamics of microbiota carriage

The dynamics of individual nasopharyngeal and oropharyngeal clusters were represented by alluvial plots and stratified by pneumococcus carriage. The number of individuals who changed clusters was reported as proportions and compared using a chi-squared test.

Temporal changes in the nasopharyngeal and oropharyngeal microbiota were analyzed at the genus level by comparing the microbiota of each individual in consecutive samples (first and second, second and third). Differences between the composition of microbiota in consecutive samples were expressed as volatility (Aitchison distance), calculated using the Euclidean distance on the CLR transformed data [34]. The Wilcoxon rank-sum test was used to compare volatility values.

## Results

### Study population

The records of 87 individuals who were followed-up for 6 months were reviewed retrospectively. Fifty-nine individuals met the following criteria to be included in the current study: 12 pneumococcal carriers with at least three samples (collected 1 month apart from each other) positive for pneumococci and 47 pneumococcal noncarriers (negative for pneumococci on at least five occasions separated 1 month apart from each other). None of the individuals included had samples collected within 1 month of antibiotic use.

The baseline characteristics of the study population are summarized in Table 1. There were no significant differences between carriers and noncarriers when mean age, sex, smoking status, and antibiotic consumption in the previous six months were compared. Pneumococcal carriers were more likely to have regular contact with children than nonpneumococcal carriers (83.3% vs. 38.3%, *P* = 0.014) and to have received seasonal flu vaccination (33.2% vs. 7.4%, *P* = 0.015). Antibiotic consumption prior to sample collection occurred in a minority of samples (and always in a period exceeding 1 month from sample collection), with no significant differences between carriers and noncarriers (Table 1).

**Table 1:** Baseline characteristics of the study population stratified by the presence/absence of pneumococci

Characteristics	Carriers *N* = 12	Noncarriers *N* = 47	*P*-value^a^
Mean age ± standard deviation (years)	36.3 ± 5.7	37.5 ± 7.1	0.142^b^
Gender male, *n* (%)	5 (41.7)	22 (46.8)	1.000
Living with children $\le$ 18 years old, *n* (%)	10 (83.3)	18 (38.3)	**0.014**
Smoker, *n* (%)	5 (41.7)	20 (42.6)	1.000
Chronic diseases, *n* (%)	6 (50.0)	12 (25.5)	0.158
Seasonal flu vaccination, *n* (%)	4 (33.4)	2 (7.4)	**0.015**
Vaccination with PCV13, *n* (%)	4 (33.4)	6 (12.8)	0.189
Antibiotic consumption 6 months before enrollment,* n* (%)	3 (25)	8 (17)	0.679

Characteristics with a *P*-value less than 0.05 are highlighted in bold

^a^Individual characteristics (with the exception of mean age) were compared with Pearson’s chi-squared test

^b^Mean age between groups was compared with Student’s *t*-test

 .

**Table 2:** Characteristics of samples included in the study according to individuals’ pneumococcal carrier state

Characteristics		Pneumococcal carriers' samples *N* = 72	Pneumococcal noncarriers' samples *N* = 282	*P*-value^a^
Antibiotic consumption between sampling, *n* (%)		6 (8.3)	20 (7.1)	0.799
Sampling season, *n* (%)	Spring	22 (30.6)	80 (28.4)	0.826
	Summer	18 (25.0)	78 (27.7)	0.761
	Autumn	6 (8.3)	50 (17.7)	0.077
	Winter	26 (36.1)	74 (26.2)	0.130

^a^Sample characteristics were compared with Pearson’s chi-squared test

### Samples analyzed

For each of the 59 individuals, paired samples collected from the oropharynx and the nasopharynx at three time points were analyzed, resulting in a total of 354 samples. Antibiotic consumption between visits occurred 8.3% of the time.﻿ Samples were collected throughout the year with no significant differences between pneumococcal carriers and noncarriers by sampling season (Table 2).

### Bacterial DNA quantification, processing of 16S rRNA gene data, and identification of clusters

**Fig. 1:**
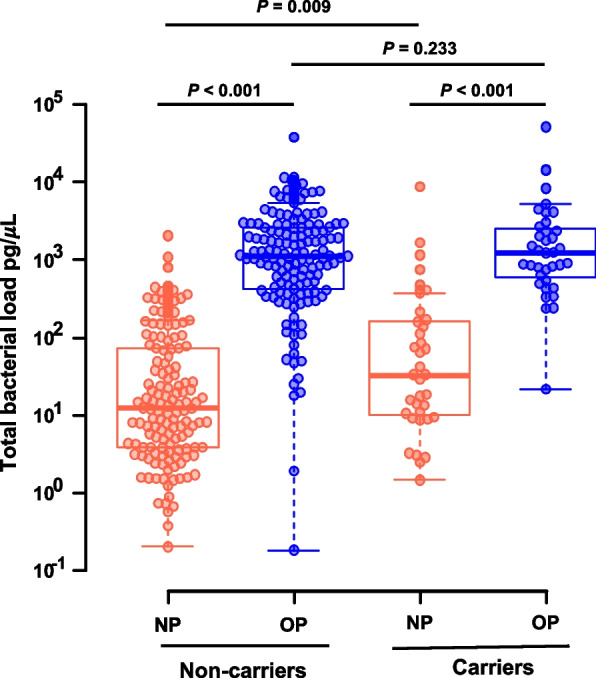
Total bacterial load of nasopharyngeal (NP) and oropharyngeal (OP) samples. The Wilcoxon rank-sum test with continuity correction and the Benjamini–Hochberg procedure were used to adjust for the false discovery rate

The geometric mean of the total bacterial load based on 16S rRNA gene quantification in the nasopharynx (19.58 pg/μL) was significantly lower (*P* < 0.001) than the bacterial DNA quantity in the oropharynx (961.27 pg/μL) independent of the pneumococcal carrier state. In the nasopharynx, the geometric mean of the total bacterial load of pneumococcal carriers was higher than that of noncarriers (43.91 pg/μL vs. 15.89 pg/μL, *P* < 0.009). In the oropharynx, the corresponding numbers were 1237.57 pg/μL (carriers) and 901.23 pg/μL (noncarriers, *P* = 0.233) (Fig. 1).

Processing of the raw metagenomic sequencing data was performed for all 354 samples as detailed in the supplementary information (“Processing of raw metagenomic sequencing data” and Fig. S1 therein).

A total of 9,027,200 reads were received. The average number of reads per sample was 22,071 (range 2 and 38,536), which were clustered in 14,669 ASVs. After removing sequences from Eukarya and Archaea, there were a total of 8,079,020 reads with a medium number of 24,652 reads per sample (range between 2 and 38,536) and 187 singletons. The SILVA database assigned 6,589 ASVs to bacteria. A total of 108 reads were unassigned, with the majority (105 reads) sampled from the nasopharynx.

**Fig. 2:**
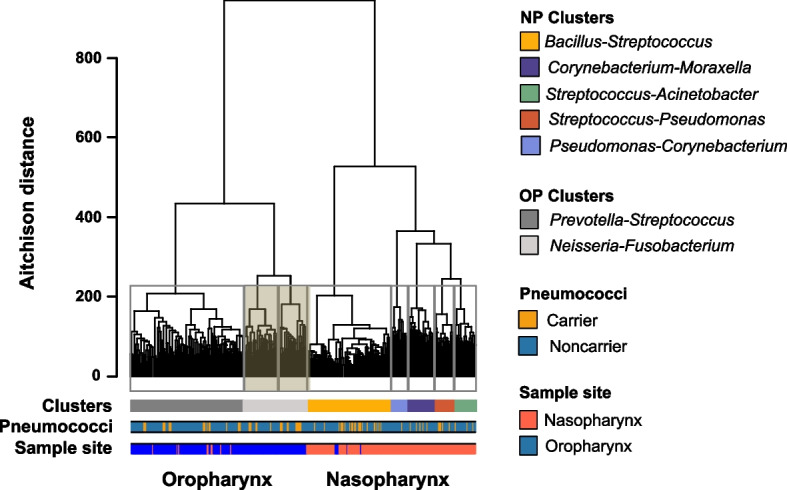
Oropharynx and nasopharynx microbiota profiles. Dendrogram showing the clusters identified by hierarchical cluster analysis performed on the Euclidean distance of the centered log-ratio transformed data (Aitchison distance). The gray lines surrounding clades represent the clusters identified by the Calinski and Harabasz index. Clusters inside the gray rectangle are the oropharyngeal clusters merged after cross-validation by random forest analysis. Bars below the dendrogram indicate clusters, samples in which pneumococcus was identified, and sampling site

To identify groups of samples that shared closer bacterial taxonomic profiles with each other, hierarchical clustering was performed. Two main groups were identified, and these, with few exceptions, segregated nasopharyngeal samples from oropharyngeal samples (Fig. 2).

A total of eight clusters were determined. However, the random forest that we used as a cross-validation method showed a confusion matrix with an out-of-bag error of 15.0% due to an overlap between two oropharyngeal clusters, where 71.4% of the samples that belonged to one of the clusters were classified in another cluster. When analyzed further, we found that the ten most abundant genera in both clusters were identical; thus, we opted to merge these two clusters. Ultimately, five nasopharyngeal clusters and two oropharyngeal clusters were observed (Fig. 2). PERMANOVA indicated that the oropharynx microbiota was significantly different from the nasopharynx microbiota (*P* < 0.001), as detailed in the supplementary information (“Bacterial profiles in the oropharynx and nasopharynx” and Fig. S2–S3 and Table S1 therein).

### Characterization of the nasopharyngeal microbiota profiles

**Fig. 3:**
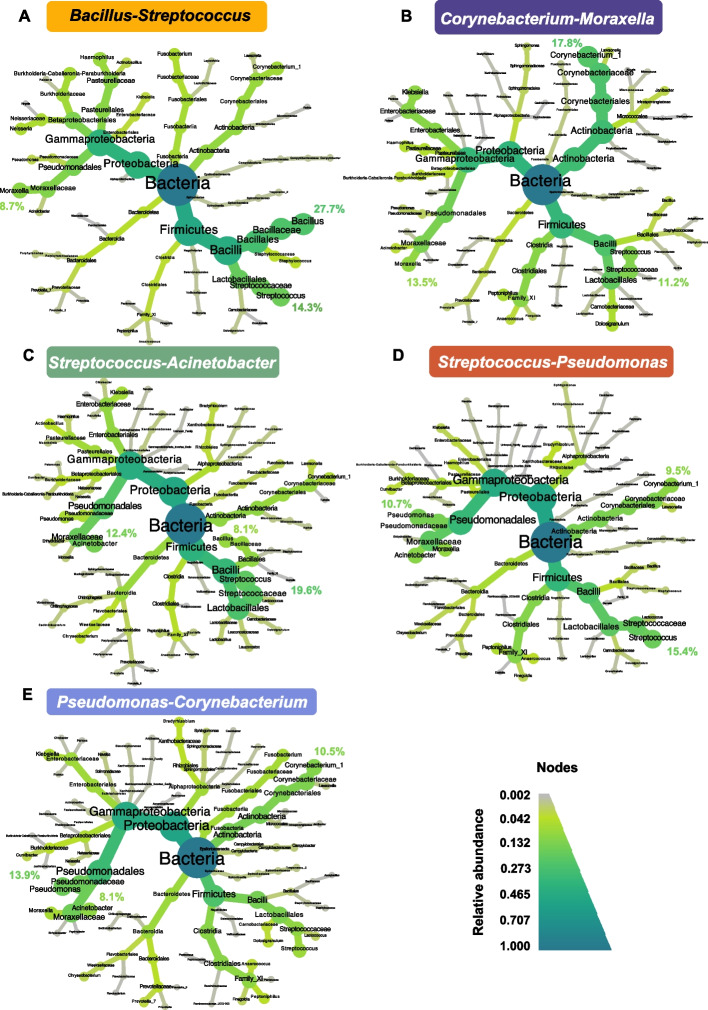
Nasopharyngeal microbiota clusters. Taxonomic heat trees for the five clusters identified. **A** *Bacillus*-*Streptococcus* (48.2% of the total samples), **B** *Corynebacterium*-*Moraxella* in dark blue (16.3%), **C** *Streptococcus-Acinetobacter* (13.3%), **D** *Streptococcus*-*Pseudomonas* (12.0%), and **E** *Pseudomonas-Corynebacterium* (10.2%). The center of each tree represents the kingdom and has a relative abundance of 1. In the extremities, the relative abundances at the genus level are represented. From the center to the extremities, each taxonomic level from kingdom to genus is indicated. The gradient of colors represents relative abundance. In each cluster, the relative abundance of the three most common genera is specified

We identified five clusters in the nasopharynx and named them after the two most abundant genera found in each cluster. The most frequent cluster was named *Bacillus*-*Streptococcus* and included 48.2% (*n* = 80) of the total samples from the nasopharynx. The three most abundant genera in this cluster were *Bacillus* (27.7%), *Streptococcus* (14.3%), and *Moraxella* (8.7%) (Fig. 3A). This cluster was the least diverse and least even of all nasopharyngeal clusters (Fig. S4A). The other clusters were named *Corynebacterium*-*Moraxella* (16.3%, *n* = 27), *Streptococcus-Acinetobacter* (13.3%, *n* = 22), *Streptococcus*-*Pseudomonas* (12.0%, *n* = 20), and *Pseudomonas-Corynebacterium* (10.2%, *n* = 17) (Fig. 3B–E).

Clusters in which *Streptococcus* were not dominant had a higher effective number of genera (supplementary information “Bacterial profiles in the oropharynx and nasopharynx” and Fig. S4 therein).

### Association between nasopharyngeal microbiota profiles and variables under study

A mixed general linear model was used to investigate potential associations between the nasopharyngeal microbiota profiles and the pneumococcal carrier state and sociodemographic and environmental characteristics. The cluster *Bacillus-Streptococcus* was used as a reference since this cluster accounted for the highest number of samples (Table 3). In winter, the nasopharyngeal microbiota was less likely to be described by the *Streptococcus-Acinetobacter* cluster (*OR* = 0.18; 95% *CI* 0.02–0.85) and more likely to be described by the *Corynebacterium-Moraxella* cluster (*OR* = 28.1, 95% *CI* 4.4–307.2) compared to the reference. Males were more likely to have a nasopharyngeal microbiota described by clusters *Corynebacterium-Moraxella* (*OR* = 227.0, 95% *CI* 25.2–679.6) or *Pseudomonas-Corynebacterium* (*OR* = 12.5, 95% *CI* 1.5–310.3). Having contact with children increased the likelihood of having a nasopharyngeal microbiota described by the cluster *Pseudomonas-Corynebacterium* (*OR* = 15.6, 95% *CI* 1.8–376.1), whereas being a smoker decreased the likelihood of having that cluster (*OR* = 0.13, 95% *CI* 0.02–0.72). Of note, *Pseudomonas-Corynebacterium* included only one pneumococcal carrier (Table 3), which may indicate that this microbiota profile has a protective role against pneumococcal carriage.

**Table 3:** Association between nasopharyngeal microbiota profiles and variables under study

Characteristics	Microbiota profiles								
	*Bacillus-Streptococcus* *N* = 80	*Streptococcus-Pseudomonas* *N* = 20		*Streptococcus-Acinetobacter* *N* = 22		*Corynebacterium-Moraxella* *N* = 27		*Pseudomonas-Corynebacterium* *N* = 17	
	*n* (%)	*n* (%)	*OR*_adj_ (95% *CI*)	*n* (%)	*OR*_adj_ (95% *CI*)	*n* (%)	*OR*_adj_ (95% *CI*)	*n* (%)	*OR*_adj_ (95% *CI*)
Pneumococcal carrier, yes	21 (26.3)	3 (18.7)	0.26 (0.04–1.21)	3 (18.7)	0.22 (0.03–1.18)	5 (18.5)	0.71 (0.04–10.59)	1 (5.8)	NA
Age, > 37 years	43 (53.7)	9 (20.0)	0.64 (0.14–2.62)	9 (20.0)	0.58 (0.15–2.06)	7 (25.9)	0.68 (0.10–3.89)	8 (47.1)	1.08 (0.12–10.15)
Gender, male	22 (27.5)	6 (37.5)	1.19 (0.35–3.77)	6 (37.5)	0.74 (0.21–2.40)	26 (96.3)	**226.97 (25.16**–**679.59)**	14 (82.6)	**12.52 (1.51**–**310.27)**
Children, yes	41 (52.2)	7 (43.7)	4.70 (1.04–25.82)	7 (43.7)	0.60 (0.15–2.39)	7 (25.9)	0.24 (0.02–1.99)	10 (58.8)	**15.59 (1.83**–**376.07)**
Smoker, yes	39 (48.7)	7 (43.7)	0.56 (0.16–1.81)	7 (43.7)	0.41 (0.12–1.23)	11 (40.7)	0.65 (0.12–3.26)	4 (23.5)	**0.13 (0.02**–**0.72)**
Season									
Spring	28 (35.0)	12 (75.0)	Ref	12 (75.0)	Ref	3 (11.1)	Ref	0 (0.0)	Ref
Summer	22 (27.5)	8 (50.0)	3.54 (0.94–15.86)	8 (50.0)	0.78 (0.23–2.53)	2 (7.4)	0.50 (0.04–4.75)	4 (23.5)	NA
Autumn	11 (13.7)	0 (0.0)	2.55 (0.38–16.32)	0 (0.0)	NA	2 (7.4)	0.71 (0.06–7.38)	12 (70.1)	NA
Winter	19 (23.8)	2 (12.5)	1.11 (0.21–5.76)	2 (12.5)	**0.18 (0.02**–**0.85)**	20 (74.1)	**28.1 (4.38**–**307.16)**	1 (5.9)	NA

*Ref*, variable used as reference. *NA,* nonadmissible as there are no data in the reference. Bold indicates statistically significant results. The *Bacillus-Streptococcus* profile was the most frequent and thus was used as a reference against which the other profiles were compared

### Nasopharyngeal profiles of subpopulations of pneumococcal carriers, adults who have close contact with children, and smokers

**Fig. 4:**
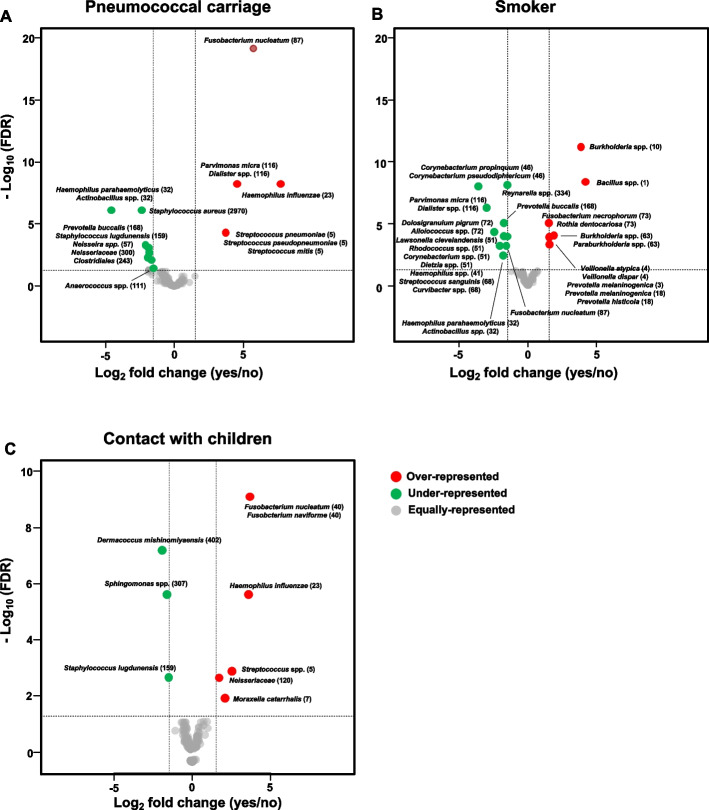
Volcano plots representing ASVs that showed differential abundance in the nasopharynx. **A** Effect of pneumococcal carrier status. **B** Effect of smoking status. **C** Effect of having contact with children. Bacterial taxa overrepresented among pneumococcal carriers, smokers, and adults who have regular contact with children are represented by red circles on the right side of each corresponding plot. Bacterial taxa underrepresented among pneumococcal carriers, smokers, and adults who have regular contact with children are represented by green circles on the left side of each corresponding plot. Gray circles indicate bacterial taxa that were not differentially abundant

To identify which ASVs differed between the nasopharyngeal microbiota based on the pneumococcal carrier state, smoking habits, and contact with children, ZIGM models were fitted. The nasopharyngeal microbiota between individuals identified as pneumococcal carriers and noncarriers showed significant differences (PERMANOVA, *P* = 0.001). Among pneumococcal carriers, four ASVs were found to be overrepresented (Fig. 4A, Table S2). These were identified as presumptive *H. influenzae* (ASV23), *Fusobacterium nucleatum* (ASV87), *Parvimonas micra* or *Dialister* spp. (ASV116), and *S. pneumoniae*, *S. pseudopneumoniae*, or *S. mitis* (ASV5). In addition, eight ASVs were found to be underrepresented. These were identified as presumptive *Haemophilus parahaemolyticus* or *Actinobacillus* spp. (ASV32), *Staphylococcus lugdunensis* (ASV159), and *Staphylococcus aureus* (ASV2970).

The nasopharyngeal microbiota between individuals identified as smokers and nonsmokers also showed significant differences (PERMANOVA, *P* = 0.001). Among smokers, seven ASVs were found to be overrepresented (Fig. 4B, Table S3). Of these, the three with the highest FCs were ASV1 (log_2_FC = 4.19), ASV10 (log_2_FC = 3.85), and ASV63 (log_2_FC = 1.88). These were identified as presumptive *Bacillus* spp. (ASV1), *Burkholderia* spp. (ASV10), and *Burkholderia* spp. or *Paraburkholderia* spp. (ASV63). Ten ASVs were found to be underrepresented. Of these, ASV41 (log_2_FC =  − 2.05), ASV46 (log_2_FC =  − 3.64), and ASV72 (log_2_FC =  − 2.46) showed the lowest FCs (Fig. 4B, Table S3). These were identified as presumptive *H. parahaemolyticus* or *Haemophilus sputorum* (ASV41), *Corynebacterium propinquum* or *Corynebacterium pseudodiphtericum* (ASV46), and *D. pigrum* or uncultured *Alloiococcus* spp. (ASV72).

Finally, differences between the nasopharyngeal microbiota of adults who had regular contact with children compared to adults who did not have frequent contact with children were also significant (PERMANOVA, *P* = 0.04). Among adults who had contact with children, five ASVs were overrepresented (Fig. 4C, Table S4). Of these, ASV5 (log_2_FC = 2.57), ASV23 (log_2_FC = 3.61), and ASV40 (log_2_FC = 3.69) had the highest FCs. The latter was identified as presumptive *F. nucleatum* or *Fusobacterium naviforme*. ASV159 (log_2_FC =  − 1.51), ASV307 (log_2_FC =  − 1.62), and ASV402 (log_2_FC =  − 1.94) were found to be underrepresented. These were identified as presumptive *S. lugdunensis* (ASV159), *Sphingomonas* spp. (ASV307), and *Dermacoccus nishinomiyaensis* (ASV402).

### Diversity of nasopharyngeal profiles of subpopulations of pneumococcal carriers, adults who have close contact with children, and smokers

**Fig. 5:**
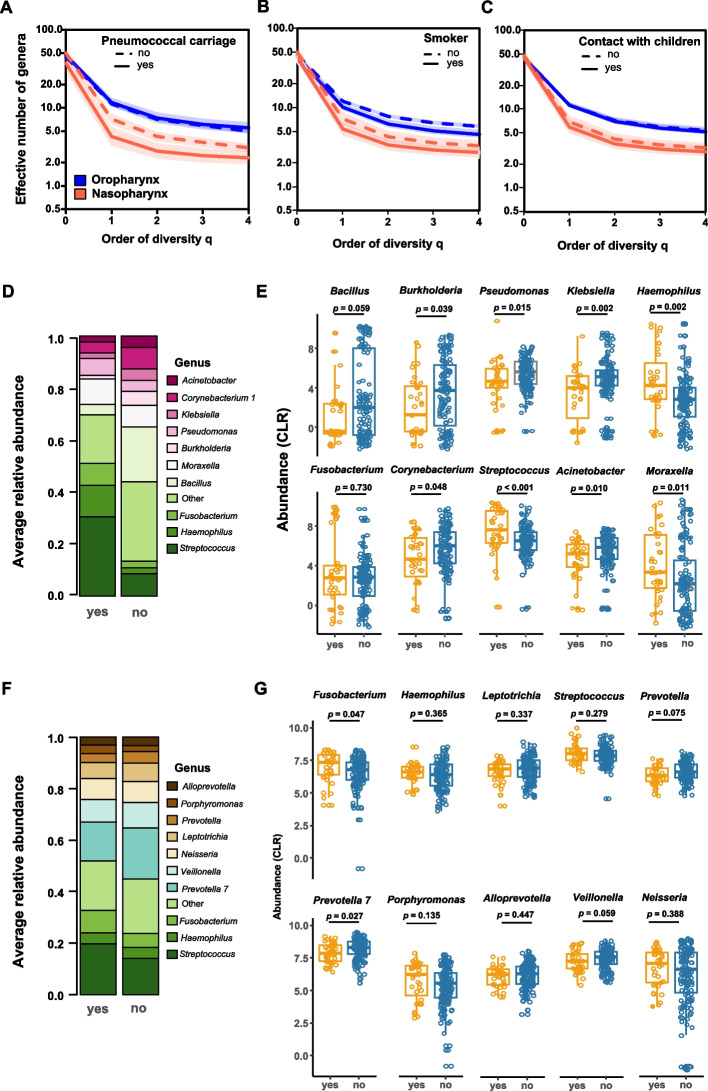
Diversity profiles. **A** Diversity of nasopharyngeal and oropharyngeal microbiota given by Hill numbers of order 0 to 4 in pneumococcal carriers and noncarriers. **B** Diversity of nasopharyngeal and oropharyngeal microbiota given by the Hill numbers of order 0 to 4 of smokers and nonsmokers. **C** Diversity of nasopharyngeal and oropharyngeal microbiota given by the Hill numbers of order 0 to 4 of individuals who have regular contact with children and those who do not have. **D** Average relative abundance of the ten most frequent genera found in the microbiota of the nasopharynx represented by stacked bar plots. The remaining less abundant genera were grouped as a single bar (other). **E** Abundance of the ten most abundant genera found in the nasopharynx of carriers and noncarriers. **F** Average relative abundance of the ten most frequent genera found in the microbiota of the oropharynx represented by stacked bar plots. The remaining less abundant genera were grouped as a single bar (other). **G** Abundance of the ten most abundant genera found in the oropharynx of carriers and noncarriers. *P*-values determined by the Wilcoxon rank-sum test

Diversity, at the taxonomic level of genus, was significantly lower in the nasopharynx of pneumococcal carriers (^0^*D* = 37.6, ^1^*D* = 4.3, ^2^*D* = 2.8) than in the nasopharynx of noncarriers (^0^*D* = 49.7, ^1^*D* = 7.1, ^2^*D* = 4.3) for each diversity number (*P* = 0.037, *P* = 0.003, and *P* = 0.006, respectively) (Fig. 5A and Fig. S5A). In addition, the nasopharynx microbiota of pneumococcal carriers was less even than the nasopharynx microbiota of noncarriers, supporting the higher dominance of the most abundant species found in this niche (Fig. S5A).

In parallel, the proportion of reads classified as *Streptococcus* in the nasopharynx of pneumococcal carriers compared to noncarriers was significantly higher (*P* < 0.001); among carriers, *Streptococcus* accounted for much as 30% on average of all genera (summing up to more than 50% of all genera in 29% of carriers); among noncarriers, *Streptococcus* accounted, on average, for 10% of all genera (summing up to more than 50% of all genera in only 3% of noncarriers) (Fig. 5D − E and Fig. S6).

Only marginal differences between the diversity exhibited by the nasopharynx of smokers (^0^*D* = 42.7, ^1^*D* = 5.4, ^2^*D* = 3.4) compared to nonsmokers (^0^*D* = 50.2, ^1^*D* = 7.3, ^2^*D* = 4.3) were found when comparing each diversity number (*P* = 0.063, *P* = 0.019, and *P* = 0.032, respectively) (Fig. 5B and Fig. S5B), and no significant differences were observed when the diversity exhibited by the microbiota of the nasopharynx of individuals who had regular contact with children (^0^*D* = 45.4, ^1^*D* = 5.9, ^2^*D* = 3.6) was compared with those without regular contact (^0^*D* = 48.4, ^1^*D* = 6.9, ^2^*D* = 4.1) (Fig. 5C and Fig. S5C).

### Characterization of oropharyngeal microbiota clusters

**Fig. 6:**
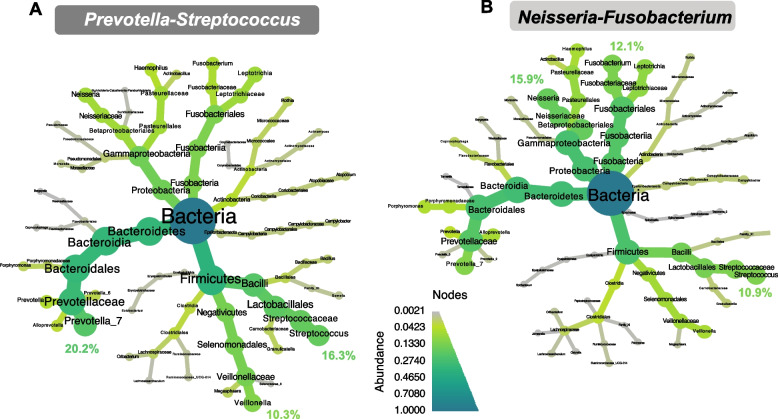
Oropharyngeal microbiota clusters. Taxonomic heat trees for the two clusters identified. **A** *Prevotella*-*Streptococcus* (83.9% of the total samples) and **B** *Neisseria*-*Fusobacterium* (16.1%). The center of each tree represents the kingdom and has a relative abundance of 1. In the extremities, the relative abundances at the genus level are represented. From the center to the extremities, each taxonomic level from kingdom to genus is indicated. The gradient of colors represents relative abundance. In each cluster, the relative abundance of the three most common genera is specified

The two oropharyngeal microbiota clusters were named *Prevotella-Streptococcus* and *Neisseria-Fusobacterium*. The *Prevotella-Streptococcus* cluster accounted for 83.9% (*n* = 151) of all samples from the oropharynx. The three most abundant genera in this cluster were *Prevotella* (20.2%), *Streptococcus* (16.3%), and *Veillonella* (10.3%) (Fig. 6A). The *Neisseria-Fusobacterium* cluster accounted for 16.1% (*n* = 29) of all samples, and the three most abundant genera were *Neisseria* (15.9%), *Fusobacterium* (12.1%), and *Streptococcus* (10.9%) (Fig. 6B). Both clusters had comparable diversity (Fig. S4B) and a high abundance of ASV2, classified as presumptive *Streptococcus* spp., which included, among other species, presumptive *S. pneumoniae* (supplementary information “Bacterial profiles in the oropharynx and nasopharynx”).

### Association between oropharyngeal microbiota profiles and variables under study

Mixed general linear models were used to investigate potential associations between the oropharyngeal microbiota profiles and the pneumococcal carrier status of the oropharynx and sociodemographic and environmental characteristics. The cluster *Prevotella-Streptococcus* was used as a reference since this cluster accounted for the highest number of samples (Table 4). Pneumococcal carriers were 3.6-fold (95% *CI* 1.3–12.1) more likely to have their oropharyngeal microbiota described by cluster *Neisseria-Fusobacterium* compared to *Prevotella-Streptococcus,* whereas smokers were 86% less likely to have their oropharyngeal microbiota described by cluster *Neisseria-Fusobacterium* (95% *CI* 0.04–0.4).

**Table 4:** Association between oropharyngeal microbiota profiles and variables under study

Characteristics	Microbiota profiles		
	*Prevotella-Streptococcus* *N* = 142	*Neisseria-Fusobacterium* *N* = 29	
	*n* (%)	*n* (%)	*OR*_adj_ (95% *CI*)
Pneumococcal carrier, yes	24 (16.9)	11 (37.9)	**3.6 (1.3**–**12.1)**
Age, > 37 years	73 (51.4)	7 (24.1)	0.4 (0.1–1.1)
Gender, male	66 (46.5)	14 (48.4)	2.1 (0.4–3.0)
Children, yes	70 (49.3)	12 (41.4)	0.6 (0.2–1.9)
Smoker, yes	67 (47.2)	4 (13.7)	**0.14 (0.04**–**0.4)**
Season			
Spring	40 (28.2)	6 (20.7)	Ref
Summer	40 (28.2)	8 (27.6)	1.56 (0.5–5.6)
Autumn	23 (16.2)	5 (17.2)	1.93 (0.4–9.0)
Winter	39 (27.4)	10 (34.5)	1.23 (0.4–4.4)

*Ref*, variable used as reference. Bold indicates statistically significant results. The *Prevotella-Streptococcus* profile was the most frequent and thus was used as a reference against which the other profile was compared

### Oropharyngeal profiles of subpopulations of pneumococcal carriers, adults who have close contact with children, and smokers

**Fig. 7:**
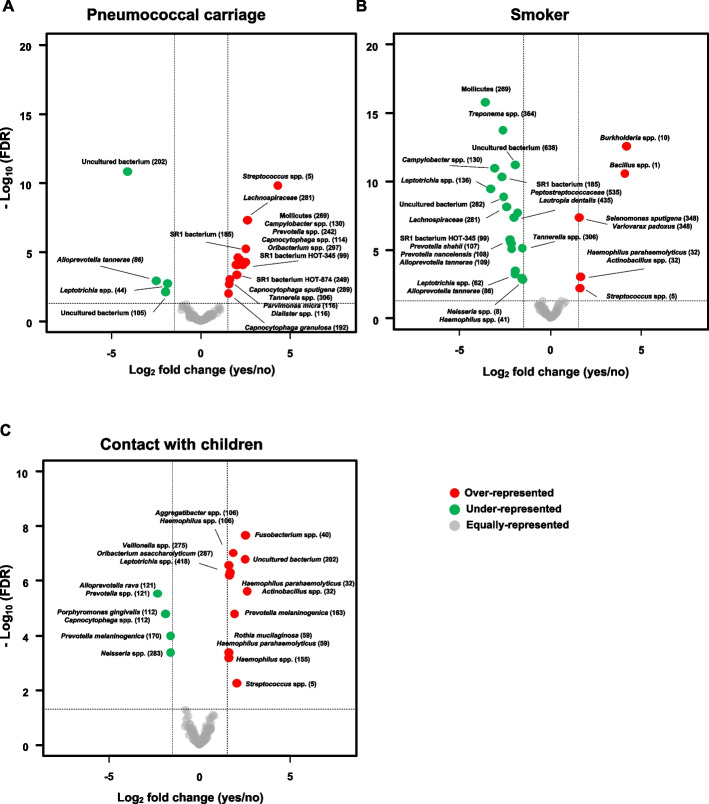
Volcano plots representing ASVs that showed differential abundance in the oropharynx. **A** Effect of pneumococcal carrier status. **B** Effect of smoking status. **C** Effect of having contact with children. Bacterial taxa overrepresented among pneumococcal carriers, smokers, and adults who have regular contact with children are represented by red circles on the right side of each corresponding plot. Bacterial taxa underrepresented among pneumococcal carriers, smokers, and adults who have regular contact with children are represented by green circles on the left side of each corresponding plot. Gray circles indicate bacterial taxa that were not differentially abundant

Significant differences between the oropharyngeal microbiota of pneumococcal carriers and nonpneumococcal carriers were found using PERMANOVA (*P* = 0.002). Eleven ASVs were overrepresented among pneumococcal carriers (Fig. 7A, Table S5), with ASV5 (log_2_FC = 4.31) and ASV281 (log_2_FC = 2.63) showing the highest FC. The latter was identified as *Lachnospiraceae*. On the other hand, ASV44 (log_2_FC =  − 1.85) and ASV86 (log_2_FC =  − 2.49) were underrepresented (Fig. 7A, Table S5). These were identified as presumptive *Leptotrichia* spp. and *Alloprevotella tannerae*, respectively.

Differences between the oropharyngeal microbiota of smokers and nonsmokers were also observed (*P* = 0.001). Five ASVs were overrepresented among smokers (Fig. 7B, Table S6). Among these, the ones with the highest FC were ASV1 (log_2_FC = 4.08) and ASV10 (log_2_FC = 4.17). Fifteen ASVs were underrepresented among smokers. Of these, ASV130 (log_2_FC =  − 3.06), ASV136 (log_2_FC =  − 3.30), and ASV269 (log_2_FC =  − 3.59) showed the lowest FCs and were identified as presumptive *Campylobacter showae* or *Campylobacter rectus*, *Leptotrichia* spp., and Mollicutes, respectively.

Finally, differences between the oropharyngeal microbiota of individuals who had regular contact with children compared to those who did not (*P* = 0.004) were observed. Ten ASVs were overrepresented in individuals who had contact with children (Fig. 7C, Table S7), with ASV32 (log_2_FC = 2.62) and ASV40 (log_2_FC = 2.54) showing the highest FC. On the other hand, four ASVs were underrepresented (Fig. 7C, Table S7): ASV112 (log_2_FC =  − 2.32), ASV121 (log_2_FC =  − 1.87), ASV170 (log_2_FC =  − 1.60), and ASV283 (log_2_FC =  − 1.60). These were identified as presumptive *Porphyromonas gingivalis* or *Capnocytophaga* spp., *Alloprevotella rava* or *Prevotella* spp., *Prevotella melaninogenica*, and *Neisseria* spp., respectively.

### Diversity of oropharyngeal profiles of subpopulations of pneumococcal carriers, adults who have close contact with children, and smokers

Diversity at the genus level was not significantly different when the oropharyngeal microbiota of pneumococcal carriers (^0^*D* = 47.3, ^1^*D* = 11.7, ^2^*D* = 7.4) and noncarriers (^0^*D* = 44.9, ^1^*D* = 11.2, ^2^*D* = 7.1) were compared (Fig. 5A and Fig. S7A). There was also no difference (*P* = 0.129) between the average proportion of reads belonging to the genus *Streptococcus* found in the oropharynx of carriers (19.8%) vs. noncarriers (14.4%) (Fig. 5F − G and Fig. S6).

In contrast, the oropharynx of smokers (^0^*D* = 43.5, ^1^*D* = 10.2, ^2^*D* = 6.3) was significantly less diverse than the oropharynx of nonsmokers (^0^*D* = 46.7, ^1^*D* = 12.2, ^2^*D* = 7.8) when comparing each diversity number (*P* = 0.095, *P* = 0.010, and *P* = 0.010, respectively) (Fig. 5B and Fig. S7B). Finally, no significant differences were observed between the oropharyngeal microbiota of individuals who had regular contact with children (^0^*D* = 45.1, ^1^*D* = 11.3, ^2^*D* = 7.0) and those who did not (^0^*D* = 45.6, ^1^*D* = 11.3, ^2^*D* = 7.2) (Fig. 5C and Fig. S7C).

### Dynamics of microbiota carriage

**Fig. 8:**
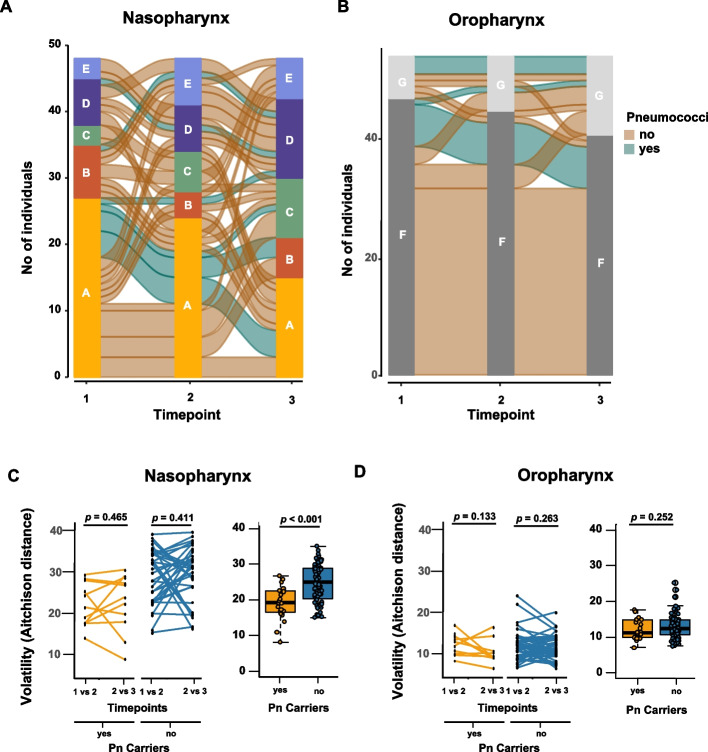
Dynamics of the nasopharyngeal and oropharyngeal microbiota. **A** Alluvial plot representing the change in individuals’ nasopharyngeal clusters, stratified by carriage of pneumococcus, at the three sampling times. **B** Alluvial plot representing the change in individuals’ oropharyngeal clusters, stratified by carriage of pneumococcus, at the three sampling times. **C** Volatility of the nasopharyngeal microbiota depending on pneumococcal carrier state. **D** Volatility of the oropharyngeal microbiota depending on pneumococcal carrier state. In **C** and **D**, left graphics show volatility per individual calculated as the Aitchison distance between microbiota at consecutive time points: first vs. second and second vs. third. Lines connect volatility values of each individual. In **C** and **D**, the graphics on the right show boxplots of aggregated volatility. Yellow indicates pneumococcal carriers; blue indicates pneumococcal noncarriers

While 71.2% of the individuals maintained the same oropharyngeal cluster across the three time points, only 15.7% maintained the same nasopharyngeal cluster (chi-square test, *P* < 0.001) (Fig. 8A − B). On average, there was a higher volatility in the nasopharynx than in the oropharynx (Fig. 8C − D). In addition, in the nasopharynx, individuals carrying pneumococci were more likely to maintain the same nasopharyngeal cluster than those not carrying pneumococci (41.6% vs. 8.8%, chi-square test, *P* = 0.015), suggesting a higher stability in the former case (Fig. 8A). This result was supported by a lower volatility of the nasopharyngeal microbiota of pneumococcal carriers vs. noncarriers (Wilcoxon rank-sum test, *P* < 0.001) (Fig. 8C). In the oropharynx, this was not observed (Fig. 8D). Other factors, such as having contact with children, being a smoker, gender, age, and season, did not impact the dynamics of carriage.

## Discussion

Few metagenomic studies focusing on adults have been published, with the majority about microbiota dysbiosis in relation to disease [15, 35, 36]. Here, we took advantage of a longitudinal study conducted among immunocompetent healthy adults aged between 25 and 50 years old [9] to study the nasopharyngeal and oropharyngeal microbiota. In addition, we also compared the nasopharynx and oropharynx microbiota based on *S. pneumoniae* carrier status, smoking habits, and regular contact with children.

We found several differences between the microbiota of the nasopharynx vs*.* the oropharynx. We observed a higher bacterial load in oropharyngeal samples and a more homogeneous microbiota across individuals with just two clusters compared to five clusters identified in the nasopharynx. These observations are in line with a study that shows that the oropharynx has a high bacterial load, and that it varies little between individuals [37].

The oropharyngeal microbiota and the nasopharynx revealed continuity and niche-specific characteristics: the bacteria thriving in the oropharynx were obligatory anaerobes (e.g., Prevotellaceae, Veillonellaceae, or Leptotrichiaceae), whereas the bacteria thriving in the nasopharynx were mostly facultative anaerobes (e.g., Moraxellaceae and Corynebacteriaceae). Streptococcaceae*,* on the other hand, were common in both sites.

In the five nasopharyngeal microbiota clusters, the genus *Streptococcus* was one of the most abundant genera in three of these clusters, and the genus *Corynebacterium* was the most abundant in the remaining two clusters. Of note, although these genera showed high abundances, they were never equally abundant in the same cluster. This result agrees with the observation of antagonistic relationships between species of these genera. For example, it has been shown that *C. accolens* is able to produce lipases and modify triacylglycerols present in the human skin, including the human nostrils, into free fatty acids, thus inhibiting the growth of *S. pneumoniae* [38]. A healthy nasopharyngeal microbiota has been frequently associated with *Corynebacterium, Dolosigranulum*, and/or *Moraxella*-dominated profiles [39–41], which coincides with two of the identified clusters in this study: cluster *Corynebacterium*-*Moraxella* and cluster *Pseudomonas*-*Corynebacterium,* which, together, were observed in 26.5% of the samples identified in the nasopharyngeal microbiota clusters. Nonetheless, in this study, the majority (73.5%) of nasopharyngeal microbiota samples were represented by clusters codominated by *Streptococcus*. As the participants in the study were healthy individuals, this suggests a broader range of clusters associated with a healthy state. Since season (winter or summer) was associated with two clusters codominated by *Corynebacterium* and *Streptococcus*, we hypothesized that nasopharyngeal clusters may be very dynamic and may shift between clusters codominated by different genera*.* An alternation between different nasopharyngeal microbiota profiles in children due to changes associated with seasonality was previously described for healthy youth and infants, which further supports our own observations [42, 43].

In the oropharynx, we found only two microbiota clusters. The most abundant genera comprised *Prevotella*, *Streptococcus*, *Neisseria*, and *Fusobacterium*, which have already been described in the healthy oropharyngeal microbiota of adults [1, 44].

We found that the microbiota composition of the nasopharynx and oropharynx could depend on population demographic characteristics (i.e., age and gender) and/or environmental factors (i.e., smoking habits, contact with children, and season). Indeed, we found that by comparing to a reference microbiota profile, there were two out of five nasopharyngeal microbiota profiles that could be associated with pneumococcal carrier state, smoking status, contact with children, sampling season, and gender. Regarding the oropharyngeal microbiota profiles, we found that the two clusters were associated with pneumococcal carrier status, smoking habits, and age. These results are in line with previous studies that also found that demographic characteristics and environmental factors can affect the microbiota of the upper respiratory tract [1, 42].

We observed that the nasopharyngeal microbiota among pneumococcal carriers had a lower evenness than that among nonpneumococcal carriers. This raises the possibility of a specialization of bacteria able to thrive in this niche.

The presence of *S. pneumoniae* in the oropharyngeal niche seems not to disrupt the normal microbiota since the microbiota of both carriers and noncarriers are very similar. As reported, the oropharynx showed higher bacterial diversity, and as an ecosystem, a higher diversity contributes to niche stability [45].

There are several reports of a synergistic relationship between pneumococcus and *H. influenzae* [46]*.* Both bacteria are part of the nasopharyngeal niche of healthy humans. However, these are also pathobionts that can cause several infections, such as bronchitis, pneumonia, otitis media, septicemia, and meningitis. To date, it is not yet known whether this interaction is strain and/or serotype-specific or their molecular mechanisms [47]. Cope et al. [48] showed that biofilms with both species had higher cell densities, and that these bacteria can modulate each other’s virulence gene expression, leading to a persistent biofilm. Aside from this interaction, Horiuchi et al*.* [49] also reported a synergistic interaction between *P. micra* and *F. nucleatum*. This type of interaction may explain the increased abundance of these specific ASVs in pneumococcal carriers.

On the other hand, the nasopharyngeal microbiota of the nonpneumococcal carriers also showed several bacterial taxa that were overrepresented or even unique in the nasopharyngeal microbiota. Among these were *Neisseria* spp. (ASV24, ASV57, and ASV131), *S. aureus* (ASV2970), and *S. lugdunensis* (ASV159), for example. Several reports have observed a negative relationship between pneumococcus and *S. aureus* and identified mechanisms possibly associated with it [50, 51]. Additionally, Brozyna et al. observed that *S. aureus* is able to enhance the growth of *S. lugdunensis* [52].

Along with *Streptococcus* (ASV5), we found other bacterial taxa associated with the oropharyngeal microbiota of pneumococcal carriers. These were either Lachnospiraceae (ASV281) or Mollicutes (ASV269), which are frequently identified as being part of a healthy microbiota [53]. However, *Capnocytophaga* (ASV114 and ASV192), *Oribacterium* (ASV297), and *Tannerella* (ASV306) were also detected. These genera are frequently associated with diseases such as periodontitis [54].

The oropharyngeal microbiota of nonpneumococcal carriers comprised a higher abundance of genera such as *Alloprevotella* (ASV86) and *Leptotrichia* (ASV44)*.* The first was found in the human oral cavity [55], whereas the latter was found to be present in the oropharynx of healthy adults [13, 56].

We have also looked for differences in the upper respiratory tract microbiota based on two population characteristics that we know from our previous study [9] to be important for pneumococcal acquisition, namely, smoking habits and having contact with children. These characteristics were associated with differences in the microbiota in both niches. Although different, both the nasopharynx and oropharynx of smokers showed high abundances of *Bacillus* (ASV1 and ASV48) and *Burkholderia* (ASV10 and ASV63). These genera comprise a high range of human pathogenic species and have been frequently associated with environmental contamination. Nonetheless, in this study, they were spread across individuals and were most likely present due to the high proportion of individuals with smoking habits in our sample. In fact, both genera have been reported as being part of the bacterial metagenome of cigarettes, providing evidence that the source of these pathogenic bacteria may be the cigarettes themselves [57]. Furthermore, we also found that *Rothia dentocariosa* (ASV73), *Prevotella melaninogenica* (ASV3 and ASV18), and *Veillonella atypica* (ASV4) were present in high abundance in the nasopharyngeal microbiota and *Selenomonas sputigena* (ASV348) in the oropharyngeal microbiota of smokers. These are all bacterial taxa usually found to be associated with oral diseases such as caries [58–61], which may be expected in smokers [62].

Finally, the upper respiratory tract microbiota of adults with contact with children was found to be different from that of adults without contact with children. For example, *Streptococcus* (ASV5), *M. catarrhalis* (ASV7), and *H. influenzae* (ASV23) were overrepresented in the nasopharyngeal microbiota of individuals who have contact with children. Interestingly, these are the most common pathobionts known to colonize the upper respiratory tract of children [18, 46]. These bacteria are capable of causing infections such as bronchitis, otitis media, sinusitis, and pneumonia in both children and adults, although they are more frequent in the first age group [1]. Thus, colonization and, consequently, infection in young adults, albeit low, may be due to the transmission of these bacteria through contact with children.

The oropharyngeal microbiota of adults with and without contact with children, as expected, was found to be mostly represented by genera already described as part of this niche [39]. Examples of these genera were *Fusobacterium* (ASV40), *Leptotrichia* (ASV418), *Haemophilus* (ASV155 and ASV106), *Veillonella* (ASV275), *Prevotella* (ASV121, ASV170 and ASV163), and *Neisseria* (ASV283). Although we observed an increase in several pathobionts in the nasopharyngeal microbiota of individuals who have regular contact with children, regarding the oropharyngeal microbiota, this increase was only noticeable for *Streptococcus* (ASV5).

In terms of dynamics, our results are in agreement with others that showed that the microbiota of the oropharynx is stable [37]. In addition, we observed that the nasopharyngeal microbiota of adults is much less stable. However, pneumococcal carriers tend to have a more stable nasopharyngeal microbiota than noncarriers. This may be the result of *S. pneumoniae* dominance, reflected by the lower evenness in the nasopharyngeal community of *S. pneumoniae* carriers.

Our study has some limitations. First, the original study aimed to investigate the dynamics of carriage of *S. pneumoniae* in immunocompetent healthy adults; therefore, STGG medium was used to store the samples. Although this may not be the ideal medium for such studies, it has been successfully used and validated previously by others [24]. Second, we were unable to use STGG as a negative control in our analyses, as no aliquots from the original study had been stored. Nevertheless, several unsupervised methods have been used to remove possible contaminants. Also, the current comparison of the upper respiratory microbiota based on the *S. pneumoniae* carrier state was performed exclusively based on the previous identification of this bacterium by culture methods and/or qPCR [1]. Finally, to meet our established criteria for the selection of individuals, we only used 12 out of 25 pneumococcal carrier individuals identified in a previous study [9] (leading to 72 paired samples out of 224 nasopharyngeal samples and 240 oropharyngeal samples). However, when taking into account the total number of individuals and samples included in this study, it is currently one of the largest studies performed in healthy adults.

Our study also has some strengths. First, to the best of our knowledge, it is a microbiota study with one of the largest number of samples from the upper respiratory tract regarding immunocompetent healthy adults. Second, it is the first study that aimed to understand the impact of *S. pneumoniae* colonization on the microbiota of both the nasopharyngeal and oropharyngeal niches.

## Conclusions

In conclusion, our study revealed notable differences between the nasopharyngeal and oropharyngeal microbiota, with the nasopharyngeal niche exhibiting lower diversity. The presence of *S. pneumoniae* in the nasopharyngeal niche led to a microbiota shift not observed at the genus level in the oropharyngeal niche. Moreover, we identified various bacterial taxa that differ in prevalence between pneumococcal carriers and noncarriers, indicating potential interactions influencing the microbiota composition. Although some of these interactions are known, there may be additional unidentified factors playing a crucial role. For instance, *P. micra* was present in both the nasopharyngeal and oropharyngeal microbiota of pneumococcal carriers, hinting at intricate relationships yet to be fully elucidated. Additionally, our study highlighted differences in the upper respiratory tract microbiota based on smoking status and contact with children. Smokers’ microbiota exhibited an excess of pathogenic bacteria often found in cigarette metagenomes and are associated with periodontal diseases. Adults with contact with children showed higher abundances of pathobionts frequently found in children, such as *Streptococcus*, *H. influenzae*, and *M. catarrhalis*.

In summary, our findings contribute to increase our understanding of how different factors shape the upper respiratory tract microbiota of adults opening the possibility of using such information to design strategies aimed to promote a healthy respiratory microbiota.

### Supplementary Information

**Additional file 1: Fig. S1.** Quality plot of the reverse and forward read sequences resulting from FastQC. Forward sequences were trimmed at position (cycle) 240 and reverse sequences were trimmed at position 230 to reach a quality score (QS) of 30 at the 25^th^ percentile. **Fig. S2.** Oropharynx and nasopharynx microbiota profiles. Barplot representing the relative abundances of the taxonomic level Class in the oropharynx and nasopharynx and of samples misclassified in each site. **Fig. S3.** Identification of balances between groups of taxa associated with discrimination of the oropharynx and nasopharynx. The components defining the selected balance are specified on top of the boxplot that represents the distribution of the balance score for each of the groups. On the right the Receiver Operator Curve (ROC) with its AUC value and the density curve for each group is shown. TPR indicates true positive rate and FPR indicates false positive rate. **Fig. S4.** Diversity profiles of nasopharyngeal and oropharyngeal clusters. **Fig. S5.** Diversity of nasopharyngeal microbiota, for each Hill number. A. Comparison between pneumococcal carriers and non-carriers. B. Comparison between smokers and non-smokers. C. Comparison between individuals who have contact with children with adults who do not have contact. **Fig. S6.** Ten most abundant genera in the nasopharynx and oropharynx depending on pneumococcal carrier state. **Fig. S7.** Alpha diversity for the oropharyngeal microbiota calculated with the Hill numbers. A according to their pneumococcal carriage status. B. smoking and C contact with children. **Table S1.** Differences in abundance at the taxonomic level of family between nasopharynx and oropharynx. **Table S2.** Bacteria (ASV) differentially present in the nasopharyngeal microbiota of pneumococcal carriers and non-carriers. **Table S3.** Bacteria (ASV) differentially present in the nasopharyngeal microbiota of smokers and non-smokers. **Table S4.** Bacteria (ASV) differentially present in the nasopharyngeal microbiota of individuals that have and do not have regular contact with children. **Table S5.** Bacteria (ASV) differentially present in the oropharyngeal microbiota of pneumococcal carriers and non-carriers. **Table S6.** Bacteria (ASV) differentially present in the oropharyngeal microbiota of smokers and non-smokers. **Table S7.** Bacteria (ASV) differentially present in the oropharyngeal microbiota of individuals that have and do not have regular contact with children.

## Data Availability

Metagenomic sequencing data as well as meta information were deposited at NCBI’s Sequence Read Archive under the BioProject accession number PRJNA962709.

## Abbreviations

ASV: Amplicon sequence variant
AUC: Area under the ROC curve
CI: Confidence interval
CLR: Centered log_10_ ratio
Ct: Cycle threshold
SD: Standard deviation
FC: Fold change
FPR: False-positive rate
OR: Odds ratio
PERMANOVA: Permutational analysis of variance
qPCR: Real-time polymerase chain reaction
ROC: Receiver operating characteristic
STGG: Skim milk, tryptone, glucose, and glycerin
TPR: True-positive rate
URT: Upper respiratory tract
WHO: World Health Organization
ZIGMM: Zero-inflated Gaussian mixed model
