# Supplementary material for: The upper respiratory tract microbiota of healthy adults is affected by Streptococcus pneumoniae carriage, smoking habits, and contact with children
Source: Microbiome. 2023 Sep 2;11:199. doi: 10.1186/s40168-023-01640-9 (PMC10474643; doi:10.1186/s40168-023-01640-9)
Supplement: Supplementary file 2 — Additional file 1: Fig. S1. Quality plot of the reverse and forward read sequences resulting from FastQC. Forward sequences were trimmed at position (cycle) 240 and reverse sequences were trimmed at position 230 to reach a quality score (QS) of 30 at the 25th percentile. Fig. S2. Oropharynx and nasopharynx microbiota profiles. Barplot representing the relative abundances of the taxonomic level Class in the oropharynx and nasopharynx and of samples misclassified in each site. Fig. S3. Identification of balances between groups of taxa associated with discrimination of the oropharynx and nasopharynx. The components defining the selected balance are specified on top of the boxplot that represents the distribution of the balance score for each of the groups. On the right the Receiver Operator Curve (ROC) with its AUC value and the density curve for each group is shown. TPR indicates true positive rate and FPR indicates false positive rate. Fig. S4. Diversity profiles of nasopharyngeal and oropharyngeal clusters. Fig. S5. Diversity of nasopharyngeal microbiota, for each Hill number. A. Comparison between pneumococcal carriers and non-carriers. B. Comparison between smokers and non-smokers. C. Comparison between individuals who have contact with children with adults who do not have contact. Fig. S6. Ten most abundant genera in the nasopharynx and oropharynx depending on pneumococcal carrier state. Fig. S7. Alpha diversity for the oropharyngeal microbiota calculated with the Hill numbers. A according to their pneumococcal carriage status. B. smoking and C contact with children. Table S1. Differences in abundance at the taxonomic level of family between nasopharynx and oropharynx. Table S2. Bacteria (ASV) differentially present in the nasopharyngeal microbiota of pneumococcal carriers and non-carriers. Table S3. Bacteria (ASV) differentially present in the nasopharyngeal microbiota of smokers and non-smokers. Table S4. Bacteria (ASV) differentially present in the nasopharyng [file 40168_2023_1640_MOESM1_ESM.docx]

**Supplementary information**

***Streptococcus pneumoniae* carriage, smoking habits and contact with children have an impact on the upper respiratory tract microbiota of healthy adults**

A Cristina Paulo^1*&^, João Lança^1&^, Sónia T Almeida^1^, Markus Hilty^2^, and Raquel Sá-Leão^1*^

**^1^**Instituto de Tecnologia Química e Biológica António Xavier, Universidade Nova de Lisboa, Oeiras, Portugal, **^2^**Institute for Infectious Diseases, Faculty of Medicine, University of Bern, Bern, Switzerland

**Sample collection**

The current study was nested in prospective longitudinal study recently published [1]. Briefly, in the original study, 87 individuals, aged 25-50 years old, living in the Lisbon metropolitan area of Portugal were recruited between February 2015 and December 2016. An initial medical consultation was performed. Thoracic and pulmonary function and the maximum expiratory flow were evaluated and demographic information, smoking habits and brief medical history were obtained. All participants presented normal expiratory flow rates including smokers. Exclusion criteria included chronic obstructive pulmonary disease, diabetes, hepatic or renal disease, HIV infection, daily inhalation therapy, immunosuppression therapy, admission to a hospital within the last three months before sampling and regular contact with sick persons or patients [1].

Nasopharyngeal, oropharyngeal and saliva samples were obtained monthly from all participants. If a sample tested positive for pneumococci, the participant was sampled weekly, until two consecutive negative samples were obtained. Nasopharyngeal and oropharyngeal samples were collected using appropriate swabs, following the WHO recommendations [2]. Saliva samples were collected by spitting into a tube. All samples were stored in STGG (skim milk, tryptone, glucose and glycerin) medium and subsequently frozen in liquid nitrogen and kept at -80°C. All samples were analyzed by classical culture-based methods and real-time PCR (qPCR) targeting *lytA* (a gene that encodes for the major autolysin of pneumococcus) and *piaB* (a gene that encodes for an iron uptake ABC transporter lipoprotein), as previously described [3,4]. Overall, 25 individuals were found to be colonized with pneumococcus at least once during the study [2]. In the current study, samples and data from the original study were used.

**Processing of metagenomic sequencing raw data**

A total of 9,027,200 reads were obtained. Filters were applied to remove ASVs that were of non-bacterial origin, contaminants, and those that did not fulfill the minimum relative abundance and minimum frequency criteria. Specifically, after inspection of the quality control plots, forward sequences were trimmed at position 240 and reverse sequences were trimmed at position 230 to reach a QS30 at the 25th percentile (Fig. S1). On average, DADA2 withdrew 34.9% of the raw reads after filtering and trimming, plus 4% after denoise and an additional 7% that were identified as chimeras. After removing sequences from Eukarya and Archaea, there were a total of 8,079,020 reads with a medium number of 24,652 reads per sample (range between 2-38,536) and 187 singletons. SILVA database assigned 6,589 ASV's to Bacteria. A total of 108 reads were unassigned being the majority (105 reads) sampled from the nasopharynx. At this stage the total number of ASVs in the nasopharyngeal and oropharyngeal samples was 5716 and 1957, respectively.

To further clean-up the data, samples from the oropharynx and the nasopharynx were split. In the group of samples from the nasopharynx 141 ASVs (2.4%) were identified by decontam as contaminants. In addition, 724 ASVs (13.0%) did not meet the criteria of being present at higher than 0.1% in a minimum of two samples, and two samples had <1,000 reads (one with 2 reads and another with 274 reads). All these ASVs were, therefore, removed. Likewise, in the oropharynx 30 ASVs (1.5%) were identified by decontam as contaminants, 512 ASVs (26.2%) did not meet the criteria of being present at a frequency of 0.1% in a minimum of two samples. All were removed. No samples had <1,000 reads. The ASVs that accounted for the four most prevalent contaminants in the nasopharynx were ASV6 found in 171 samples (after blastn classified as belonging to the *Staphylococcus* genera), ASV78 found in 127 samples (*Flavobacterium* sp. or *Pantoea dispersa*), ASV208 found in 62 samples (uncultured bacterium, uncultured *Sphingomonas* sp. or uncultured *Alphaproteobacterium*) and ASV1382 found in 25 samples (*Lactococcus plantarum*, uncultured bacterium or *Lactococcus piscium* strain). In the oropharynx the four most prevalent sequences identified as contaminants were ASV102 found in 24 samples (after blastn classified as presumptive *Acinetobacter* sp., *A. dispersus* or *A. tjembergiae*), ASV28 found in 20 samples (*Corynebacterium* sp. or *C. segmentosus* or uncultured *Actinobacterium*), ASV147 found in 19 samples (uncultured bacterium, uncultured *Sphingobacteria* bacterium) and ASV138 and ASV178, found in 17 samples each, (*Acinetobacter baumannii* or *Acinetobacter* spp., and *Flavobacterium* spp. or *Pantoae* spp., respectively).

Altogether, a total of 352 samples from 59 individuals and 936 ASVs were kept for downstream analysis. This corresponded to a total of 7,489,485 reads with a medium number of 22,629.5 reads per sample (range between 1,027- 38,084).

**Figure S1. Quality plot of the reverse and forward read sequences resulting from FastQC.**

Forward sequences were trimmed at position (cycle) 240 and reverse sequences were trimmed at position 230 to reach a quality score (QS) of 30 at the 25^th^ percentile.

**Bacterial profiles in the oropharynx and nasopharynx**

The PERMANOVA indicated that the oropharynx microbiota is significantly different from the nasopharynx (*P* < 0.001). Using metagenomeSeq and a ZIGM model we found that classes (families) *Negativicutes* (*Veillonellaceae*), *Fusobacteria* (*Leptotrichiaceae*), and *Bacteroidia* (*Prevotellaceae*) were underrepresented in the nasopharynx (compared to the oropharynx) (log_2_FC < -5; *P* < 0.01). On the other hand, *Gammaproteobacteria* (*Moraxellaceae*, *Pseudomonadaceae*, *Enterobacteriaceae*, *Xanthomonadaceae*), *Alphaproteobacteria* (*Xanthobacteraceae*, *Sphingomonadaceae*, *Caulobacteraceae*, *Solimonadaceae*), *Actinobacteria* (*Corynebacteriaceae*), *Bacilli* (*Lactobacillaceae*), and *Chitinophagia* (*Chitinophagaceae*) were overrepresented in nasopharynx (log_2_FC > 5; *P* < 0.001) (Fig. S2 and Table S1).

**Figure S2.** **Oropharynx and nasopharynx microbiota profiles.** Barplot representing the relative abundances of the taxonomic level Class in the oropharynx and nasopharynx and of samples misclassified in each site.

**Table S1. Differences in abundance at the taxonomic level of family between nasopharynx and oropharynx.**

|  |  | **MetagenomeSeq** | |  |
| --- | --- | --- | --- | --- |
| **ASV** | **Taxonomy** | **Log_2_ FC** | ***P*-value_adj_** |  |
| 1 | *Bacillaceae* | 3.29 | 1.82x10^-07^ |  |
| 3 | *Prevotellaceae* | -5.56 | 5.98x10^-69^ |  |
| 4 | *Veillonellaceae* | -5.97 | 5.61x10^-87^ |  |
| 7 | *Moraxellaceae* | 5.94 | 1.23x10^-51^ |  |
| 8 | *Neisseriaceae* | -3.37 | 1.16x10^-18^ |  |
| 10 | *Burkholderiaceae* | 3.70 | 4.21X10^-22^ |  |
| 12 | *Fusobacteriaceae* | -3.75 | 3.06x10^-28^ |  |
| 14 | *Pasteurellaceae* | -3.77 | 4.77x10^-26^ |  |
| 15 | *Pseudomonadaceae* | 6.37 | 4.94x10^-53^ |  |
| 16 | *Leptotrichiaceae* | -5.83 | 3.08x10^-64^ |  |
| 19 | *Enterobacteriaceae* | 6.25 | 1.02x10^-54^ |  |
| 28 | *Corynebacteriaceae* | 5.78 | 6.01x10^-49^ |  |
| 31 | *Porphyromonadaceae* | -3.46 | 5.74x10^-30^ |  |
| 34 | *Atopobiaceae* | -4.08 | 1.18x10^-33^ |  |
| 39 | Clostridiales Family_XI | 3.72 | 8.77x10^-28^ |  |
| 65 | *Xanthobacteraceae* | 6.23 | 2.22x10^-55^ |  |
| 80 | *Lachnospiraceae* | -4.45 | 3.13x10^-67^ |  |
| 83 | *Actinomycetaceae* | -3.28 | 1.38x10^-44^ |  |
| 127 | *Leuconostocaceae* | 4.50 | 6.19x10^-41^ |  |
| 143 | *Sphingomonadaceae* | 5.62 | 1.58x10^-55^ |  |
| 147 | *Chitinophagaceae* | 5.99 | 1.71x10^-56^ |  |
| 158 | *Lactobacillaceae* | 5.03 | 1.67x10^-54^ |  |
| 159 | *Staphylococcaceae* | 4.09 | 1.10x10^-37^ |  |
| 167 | *Caulobacteraceae* | 5.70 | 1.11x10^-63^ |  |
| 219 | *Solimonadaceae* | 5.88 | 1.14x10^-60^ |  |
| 230 | *Xanthomonadaceae* | 5.87 | 1.01x10^-68^ |  |
| 236 | Unknown family | 5.27 | 4.82x10^-64^ |  |
| 246 | *Aeromonadaceae* | 5.00 | 9.75x10^-71^ |  |
| 353 | *Bacteroidaceae* | 4.32 | 3.69x10^-61^ |  |

A negative value “x” means the family is Log_2_x times less abundant in the nasopharynx. Likewise, a positive value “x” means the family is Log_2_x times more abundant in the nasopharynx.

The most discriminant taxon at the level of class were a balance between *Negativicutes* (ASV4) and the group of *Actinobacteria* (ASV28) together with *Bacilli* (ASV2) and *Clostridia* (ASV39) (Fig. S3).

At the taxonomic level of genus we observed that the microbiota of the nasopharynx is significantly less diverse (^0^*D* = 46.9, ^1^*D* = 6.4, ^2^*D* = 3.9) than the oropharynx (^0^*D* = 45.3, ^1^*D* = 11.2, ^2^*D* = 7.1) except for the ^0^*D* in which there was no significant difference (*P* = 0.169). The steepness decay in the value of effective number of genera between ^0^*D* and ^1^*D* also indicated that the nasopharynx has a significantly higher uneven community compared to the one in the oropharynx.

In the nasopharynx the most abundant bacterial genus was *Streptococcus* with a mean relative abundance of 13.9%, followed by *Bacillus* (11.8%), *Corynebacerium* (8.7%), *Moraxella* (7.3%), and *Haemophilus* (4.7%). In the oropharynx the most abundant bacteria genus were *Prevotella* (18.1%), *Streptococcus* (15.2%), *Veillonella* (9.3%), *Neisseria* (8.1%), and *Leptotrichia* (6.6%).

**Figure S3.** **Identification of balances between groups of taxa associated with discrimination of the oropharynx and nasopharynx.** The components defining the selected balance are specified on top of the boxplot that represents the distribution of the balance score for each of the groups. On the right the Receiver Operator Curve (ROC) with its AUC value and the density curve for each group is shown. TPR indicates true positive rate and FPR indicates false positive rate.

**Characterization of the nasopharyngeal and oropharyngeal microbiota clusters**

*Streptococcus* was dominant in three of the five clusters identified in the nasopharynx and was the second or third most frequent genus among the two clusters identified in the oropharynx.

Twenty-four ASVs belonging to the genera *Streptococcus* were identified of which ASV5, ASV2 and ASV21 were the most abundant in decreasing order. ASV5 was classified, after NCBI blast, as presumptive *S. pneumoniae* or *S. pseudopneumoniae* or *S. mitis*. ASV2 was identified as a presumptive *Streptococcus* spp. with the possibility of being *S. pneumoniae*. ASV21 was identified as a presumptive *Streptococcus* spp. but not as *S. pneumoniae*.

All nasopharyngeal clusters except for *Pseudomonas*-*Corynebacterium*, had a high abundance of ASV5 among the ASVs matching with *Streptococcus*. More specifically, ASV5 contributed to 81.4% of all ASVs present in *Streptococcus* genera in cluster *Bacillus-Streptococcus*; 45.1% in cluster *Streptococcus-Pseudomonas*; 53.3% in cluster *Streptococcus-Acinetobacter*; 36.1% in cluster *Corynebacterium-Moraxella*; and, 6.4% in cluster *Pseudomonas-Corynebacterium*. In this last cluster the most abundant *Streptococcus* ASV was ASV21 (49.6%). In the remaining clusters this ASV represented 2.2%-25.7% of the *Streptococcus* genus. Finally, ASV2 comprised 6.3-32.6% of all ASVs present in the *Streptococcus* genus.

Among the oropharyngeal clusters, ASV2 was the most abundant: the *Prevotella-Streptococcus* cluster had an abundance of 65.2% of ASV2, 2.1% of ASV5, and 9.0% of ASV21; the *Neisseria-Fusobacterium* cluster had an abundance of 49.8% of ASV2, 4.9% of ASV5, and 4.1% of ASV21.

Diversity, at the genus level, expressed as Hill numbers of order ^0^*D*,^1^*D* and ^2^*D* was calculated for each nasopharyngeal and oropharyngeal cluster (Fig. S4).


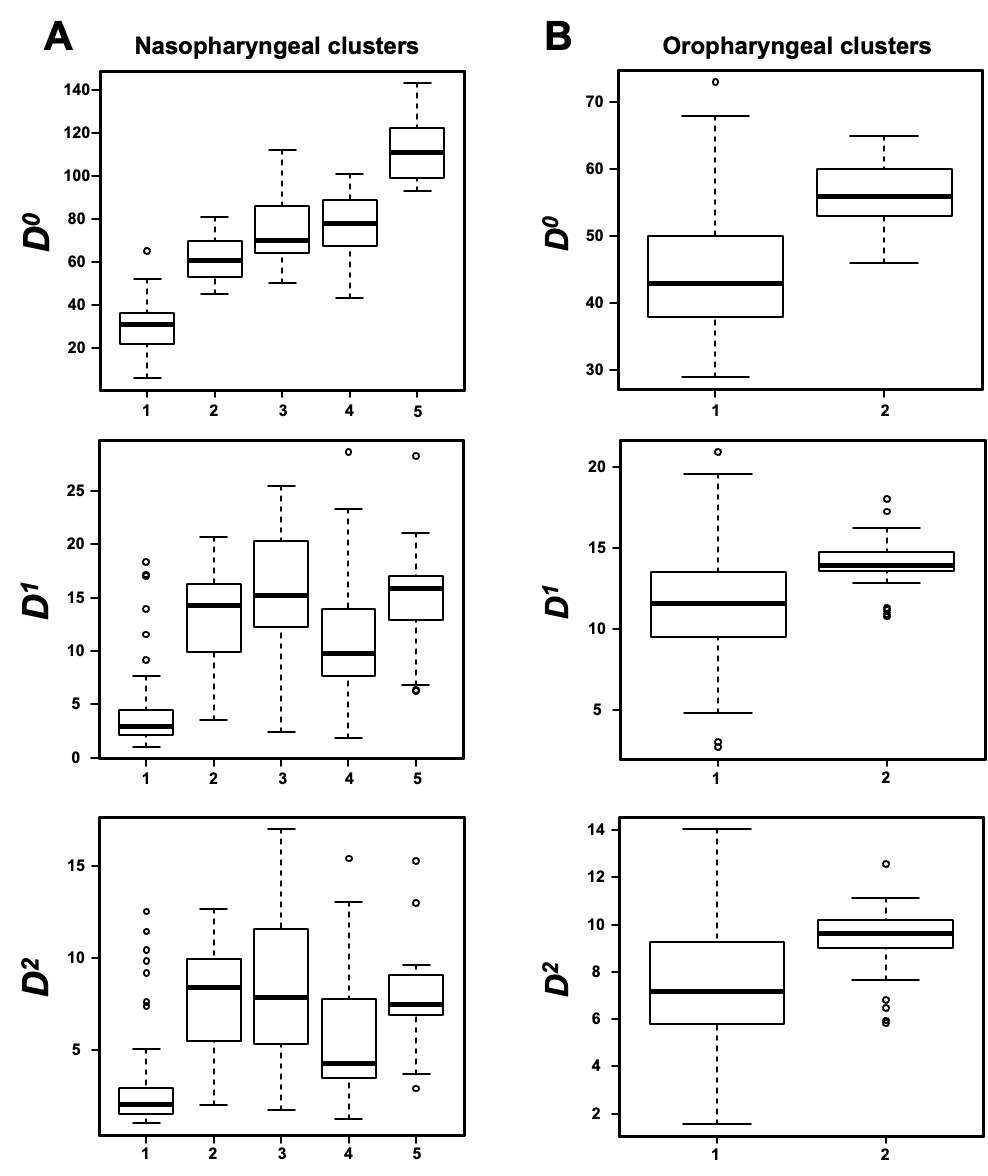


**Figure S4. Diversity profiles of nasopharyngeal and oropharyngeal clusters.**

Diversity, at the genus level, expressed as Hill numbers of order ^0^*D*,^1^*D* and ^2^*D* is shown for each bacterial cluster found in the nasopharynx (A) and oropharynx (B). Numbers 1-5 in panel A correspond to clusters *Bacillus*-*Streptococcus*, *Streptococcus-Acinetobacter*, *Streptococcus*-*Pseudomonas*, *Corynebacterium*-*Moraxella*, and *Pseudomonas-Corynebacterium*, respectively. Numbers 1 and 2 in panel B correspond to clusters *Prevotella-Streptococcus* and *Neisseria-Fusobacterium*, respectively. **A.** In the nasopharynx, cluster *Bacillus-Streptococcus* (^0^*D*=27.6, ^1^*D*=3.3, ^2^*D*=2.3) was the less diverse and uneven. Cluster *Pseudomonas-Corynebacterium* (^0^*D*=112.5, ^1^*D*=13.7, ^2^*D*=7.2) had the higher richness. Cluster *Streptococcus-Acinetobacter* (^0^*D*=72.8, ^1^*D*=13.6, ^2^*D*=7.3) did not differ from cluster *Streptococcus-Pseudomonas* (^0^*D*=60.6, ^1^*D*=12.3, ^2^*D*=6.9) and both were more diverse than cluster *Corynebacterium-Moraxella* (^0^*D*=75.4, ^1^*D*=9.5, ^2^*D*=4.9) at the Hill number of order 2. **B.** In the oropharynx, cluster *Prevotella-Streptococcus* less diverse and uneven (^0^*D*=43.9, ^1^*D*=10.9, ^2^*D*=6.8) than cluster *Neisseria-Fusobacterium* (^0^*D*=55.5, ^1^*D*=14.0, ^2^*D*=9.3).

**Nasopharyngeal profiles of subpopulations of pneumococcal carriers, adults who have close contact with children, and smokers**

**Table S2. Bacteria (ASV) differentially present in the nasopharyngeal microbiota of pneumococcal carriers and non-carriers.**

| **Carriers *vs* non-carriers** | **Log_2_ FC** | ***P*** | ***P*_adj_** |
| --- | --- | --- | --- |
| **Presumptive species after BLAST (ASV)** |  |  |  |
| *Streptococcus pneumoniae, S. pseudopneumoniae* or *S. mitis* (5) | 3.73 | < 0.001 | < 0.001 |
| *Haemophilus influenzae* (23) | 7.73 | < 0.001 | < 0.001 |
| *Fusobacterium nucleatum* (87) | 5.74 | < 0.001 | < 0.001 |
| *Parvimonas micra* or uncultured *Dialister* spp. (116) | 4.55 | < 0.001 | < 0.001 |
| *Haemophilus parahaemolyticus* or uncultured *Actinobacillus* spp. (32) | -4.78 | < 0.001 | < 0.001 |
| *Neisseria sicca* or *Neisseria mucosa* (57) | -1.68 | < 0.001 | 0.007 |
| *Anaerococcus* spp. (111) | -1.54 | 0.004 | 0.04 |
| *Staphylococcus lugdunensis* (159) | -1.88 | < 0.001 | < 0.001 |
| *Prevotella buccalis* (168) | -2.11 | < 0.001 | < 0.001 |
| Uncultured Clostridiales (243) | -1.84 | < 0.001 | 0.002 |
| Uncultured *Neisseriaceae* (300) | -1.93 | < 0.001 | 0.005 |
| *Staphylococcus aureus* (2970) | -2.38 | < 0.001 | < 0.001 |

**Table S3. Bacteria (ASV) differentially present in the nasopharyngeal microbiota of smokers and non-smokers.**

| **Smokers *vs* non-smokers** | **Log_2_ FC** | ***P*** | ***P*_adj_** |
| --- | --- | --- | --- |
| **Presumptive species after BLAST (ASV)** |  |  |  |
| *Bacillus* spp. (1) | 4.19 | < 0.001 | < 0.001 |
| *Prevotella melaninogenica* (3) | 1.54 | < 0.001 | < 0.001 |
| *Veillonella atypica* or *Veillonella dispar* (4) | 1.57 | < 0.001 | < 0.001 |
| *Burkholderia* spp*.* (10) | 3.85 | < 0.001 | < 0.001 |
| *Prevotella melaninogenica* or *Prevotella histicola* (18) | 1.56 | < 0.001 | < 0.001 |
| *Burkholderia* spp*.* or *Paraburkholderia* spp. (63) | 1.88 | < 0.001 | < 0.001 |
| *Fusobacterium necrophorum* or *Rothia dentocariosa* (73) | 1.52 | < 0.001 | < 0.001 |
| *Haemophilus parahaemolyticus* or uncultured *Actinobacillus* spp. (32) | -1.79 | 0.002 | 0.004 |
| *Haemophilus parahaemolyticum* or *Haemophilus sputorum* (41) | -2.05 | < 0.001 | < 0.001 |
| *Corynebacterium propinquum* or *Corynebacterium pseudodiphtericum* (46) | -3.64 | < 0.001 | < 0.001 |
| *Lawsonella clevelandensis,* uncultured *Dietzia* spp., uncultured *Coryne- bacterium* spp. or uncultured *Rhodococcus* spp. (51) | -1.74 | < 0.001 | < 0.001 |
| *Curvibacter gracilis, Curvibacter lanceolatus* or *Streptococcus sanguinis* (68) | -1.57 | < 0.001 | < 0.001 |
| *Dolosigranulum pigrum* or uncultured *Alloiococcus* spp. (72) | -2.46 | < 0.001 | < 0.001 |
| *Fusobacterium nucleatum* (87) | -1.69 | < 0.001 | < 0.001 |
| *Parvimonas micra* or uncultured *Dialister* spp. (116) | -1.99 | < 0.001 | < 0.001 |
| *Prevotella buccalis* (168) | -1.74 | < 0.001 | < 0.001 |
| *Reynarella* spp. (334) | -1.52 | < 0.001 | < 0.001 |

**Table S4. Bacteria (ASV) differentially present in the nasopharyngeal microbiota of individuals that have and do not have regular contact with children.**

| **Contact with children vs no contact** | **Log_2_ FC** | ***P*** | ***P*_adj_** |
| --- | --- | --- | --- |
| **Presumptive species after BLAST (ASV)** |  |  |  |
| *Streptococcus pneumoniae, S. pseudopneumoniae* or *S. mitis* (5) | 2.57 | < 0.001 | 0.001 |
| *Moraxella catarrhalis* (7) | 2.09 | 0.001 | 0.01 |
| *Haemophilus influenzae* (23) | 3.61 | < 0.001 | < 0.001 |
| *Fusobacterium nucleatum* or *Fusobacterium naviforme* (40) | 3.69 | < 0.001 | < 0.001 |
| Uncultured *Neisseriaceae* (120) | 1.73 | < 0.001 | 0.002 |
| *Staphylococcus lugdunensis* (159) | -1.51 | < 0.001 | < 0.001 |
| *Sphingomonas* spp. (307) | -1.62 | < 0.001 | < 0.001 |
| *Dermacoccus nishinomiyaensis* (402) | -1.94 | < 0.001 | < 0.001 |

**Diversity of nasopharyngeal profiles of subpopulations of pneumococcal carriers, adults who have close contact with children, and smokers.**

**Figure S5. Diversity of nasopharyngeal microbiota, for each Hill number. A.** Comparison between pneumococcal carriers and non-carriers. **B.** Comparison between smokers and non-smokers. **C.** Comparison between individuals who have contact with children with adults who do not have contact.

**Figure S6. Ten most abundant genera in the nasopharynx and oropharynx depending on pneumococcal carrier state.**

**Figure S7. Alpha diversity for the oropharyngeal microbiota calculated with the Hill numbers.** A. according to their pneumococcal carriage status. B. smoking and C contact with children.

**Table S5. Bacteria (ASV) differentially present in the oropharyngeal microbiota of pneumococcal carriers and non-carriers.**

| **Carriers *vs* non-carriers** | **Log_2_ FC** | ***P*** | ***P*_adj_** |
| --- | --- | --- | --- |
| **Presumptive species after BLAST (ASV)** |  |  |  |
| *Streptococcus pneumoniae, S. pseudopneumoniae* or *S. mitis* (5) | 4.31 | < 0.001 | < 0.001 |
| SR1 bacterium human oral taxon HOT-345 (99) | 2.36 | < 0.001 | < 0.001 |
| *Capnocytophaga* spp. (114) | 1.99 | < 0.001 | < 0.001 |
| *Parvimonas micra* or uncultured *Dialister* spp. (116) | 1.62 | < 0.001 | < 0.001 |
| *Campylobacter showae* or *Campylobacter rectus* (130) | 2.17 | < 0.001 | < 0.001 |
| Uncultured candidate division SR1 bacterium (185) | 2.53 | < 0.001 | < 0.001 |
| *Capnocytophaga granulosa* (192) | 1.55 | 0.001 | 0.01 |
| Uncultured *Prevotella* spp. (242) | 2.53 | < 0.001 | < 0.001 |
| Uncultured *Mollicutes* (269) | 2.08 | < 0.001 | < 0.001 |
| Uncultured *Lachnospiraceae* (281) | 2.63 | < 0.001 | < 0.001 |
| *Capnocytophaga sputigena* (289) | 1.59 | < 0.001 | 0.002 |
| Uncultured *Oribacterium* spp. (297) | 2.06 | < 0.001 | < 0.001 |
| *Tannerela* spp. (306) | 1.64 | < 0.001 | < 0.001 |
| *Leptotrichia* spp. (44) | -1.85 | < 0.001 | 0.002 |
| *Alloprevotella* *tannerae* (86) | -2.49 | < 0.001 | < 0.001 |
| Uncultured bacteria (105) | -1.98 | 0.001 | 0.008 |
| Uncultured bacterium (202) | -4.09 | < 0.001 | < 0.001 |

**Table S6. Bacteria (ASV) differentially present in the oropharyngeal microbiota of smokers and non-smokers.**

| **Smokers *vs* non-smokers** | **Log_2_ FC** | ***P*** | ***P*_adj_** |
| --- | --- | --- | --- |
| **Presumptive species after BLAST (ASV)** |  |  |  |
| *Bacillus* spp. (1) | 4.08 | < 0.001 | < 0.001 |
| *Streptococcus pneumoniae, S. pseudopneumoniae* or *S. mitis* (5) | 1.61 | 0.002 | 0.007 |
| *Burkholderia* spp. (10) | 4.17 | < 0.001 | < 0.001 |
| *Haemophilus parahaemolyticus* or uncultured *Actinobacillus* spp. (32) | 1.67 | < 0.001 | < 0.001 |
| *Selenomonas sputigena* or *Variovarax paradoxus* (348) | 1.58 | < 0.001 | < 0.001 |
| *Neisseria* spp. (8) | -1.57 | < 0.001 | 0.001 |
| *Haemophilus parahaemolyticus* or *Haemophilus sputorum* (41) | -1.52 | < 0.001 | 0.002 |
| Uncultured *Leptotrichia* spp. (62) | -1.95 | < 0.001 | < 0.001 |
| *Alloprevotella tannerae* (86) | -1.94 | < 0.001 | < 0.001 |
| SR1 bacterium human oral taxon HOT-345 (99) | -2.20 | < 0.001 | < 0.001 |
| *Prevotella shahii* (107) | -2.24 | < 0.001 | < 0.001 |
| *Prevotella nanceiensis* (108) | -2.14 | < 0.001 | < 0.001 |
| *Alloprevotella tannerae* (109) | -2.13 | < 0.001 | < 0.001 |
| *Campylobacter showae* or *Campylobacter rectus* (130) | -3.06 | < 0.001 | < 0.001 |
| Uncultured *Leptotrichia* spp. (136)* | -3.30 | < 0.001 | < 0.001 |
| Uncultured candidate division SR1 bacterium (185) | -2.41 | < 0.001 | < 0.001 |
| Uncultured Mollicutes (269) | -3.59 | < 0.001 | < 0.001 |
| Uncultured *Lachnospiraceae* (281) | -2.66 | < 0.001 | < 0.001 |
| Uncultured bacterium (282) | -2.58 | < 0.001 | < 0.001 |
| *Tannerella* spp. (306) | -1.54 |  |  |
| Uncultured *Treponema* spp. (364) | -2.61 | < 0.001 | < 0.001 |
| *Lautropia dentalis* (435) | -1.99 | < 0.001 | < 0.001 |
| Uncultured *Peptostreptococcaceae* (535) | -1.80 | < 0.001 | < 0.001 |
| Uncultured bacterium (638) | -1.94 | < 0.001 | < 0.001 |

* BLAST identity < 100%.

**Table S7. Bacteria (ASV) differentially present in the oropharyngeal microbiota of individuals that have and do not have regular contact with children.**

| **Contact with children vs no contact** | **Log_2_ FC** | ***P*** | ***P*_adj_** |
| --- | --- | --- | --- |
| **Presumptive species after BLAST (ASV)** |  |  |  |
| *Streptococcus pneumoniae, S. pseudopneumoniae* or *S. mitis* (5) | 1.84 | < 0.001 | 0.006 |
| *Haemophilus parahaemolyticus* or uncultured *Actinobacillus* spp. (32) | 2.62 | < 0.001 | < 0.001 |
| *Fusobacterium nucleatum* or *Fusobacterium naviforme* (40) | 2.54 | < 0.001 | < 0.001 |
| *Rothia mucilaginosa* or *Haemophilus parahaemolyticus* (59) | 1.62 | < 0.001 | < 0.001 |
| *Aggregatibacter segnis, Aggegatibacter aphrophilus* or uncultured *Haemophilus* spp. (106) | 1.86 | < 0.001 | < 0.001 |
| *Haemophilus parainfluenzae* or *Haemophilus pittmaniae* (155) | 1.61 | < 0.001 | < 0.001 |
| *Prevotella melaninogenica* (163) | 1.93 | < 0.001 | < 0.001 |
| Uncultured bacterium (202) | 2.52 | < 0.001 | < 0.001 |
| *Veillonella* spp*.* (275) | 1.70 | < 0.001 | < 0.001 |
| *Oribacterium asaccharolyticum* (287) | 1.63 | < 0.001 | < 0.001 |
| *Porphyromonas gingivalis* or uncultured *Capnocytophaga spp.* (112) | -2.32 | < 0.001 | < 0.001 |
| *Alloprevotella rava* or uncultured *Prevotella* spp*.*(121) | -1.87 | < 0.001 | < 0.001 |
| *Prevotella melaninogenica* (170)*** | -1.60 | < 0.001 | < 0.001 |
| Uncultured *Neisseria* spp. (283) | -1.60 | < 0.001 | < 0.001 |

* BLAST identity < 100%.

**References**

1. Almeida ST, Paulo AC, Froes F, de Lencastre H, Sá-Leão R. Dynamics of pneumococcal carriage in adults: a new look at an old paradigm. J Infect Dis. 2021;223(9):1590-600; doi: 10.1093/infdis/jiaa558.

2. Satzke C, Turner P, Virolainen-Julkunen A, Adrian PV, Antonio M, Hare KM, et al. Standard method for detecting upper respiratory carriage of *Streptococcus pneumoniae*: updated recommendations from the World Health Organization Pneumococcal Carriage Working Group. Vaccine. 2013;32(1):165-79; doi: 10.1016/j.vaccine.2013.08.062.

3. Trzciński K, Bogaert D, Wyllie A, Chu ML, van der Ende A, Bruin JP, et al. Superiority of trans-oral over trans-nasal sampling in detecting *Streptococcus pneumoniae* colonization in adults. PLoS One. 2013;8(3):e60520; doi: 10.1371/journal.pone.0060520.

4. Tavares DA, Handem S, Carvalho RJ, Paulo AC, de Lencastre H, Hinds J, et al. Identification of *Streptococcus pneumoniae* by a real-time PCR assay targeting SP2020. Sci Rep. 2019;9(1):3285; doi: 10.1038/s41598-019-39791-1.
